# Supplementary material for: treeclimbR pinpoints the data-dependent resolution of hierarchical hypotheses
Source: Genome Biol. 2021 May 17;22:157. doi: 10.1186/s13059-021-02368-1 (PMC8127214; doi:10.1186/s13059-021-02368-1)
Supplement: Supplementary file 1 — Additional file 1 Supplementary materials [file 13059_2021_2368_MOESM1_ESM.pdf]

Supplementary Material

Contents

1 Supplementary Figures
1.1 Schematic overview of treeclimbR on the DS case
1.2 Results of parametric synthetic microbial datasets (fixed signal branches)
1.3 Results of non-parametric synthetic microbial datasets
1.4 Results of BCR-XL-sim datasets
1.5 Benchmark methods' runtimes

2 Supplementary Tables
2.1 Results of miRNA data
2.2 Results of parametric synthetic microbial datasets (fixed signal branches)
2.3 Results of AML-sim datasets

3 Supplementary Note 1: Simulation framework
3.1 Balanced signal: BS
3.2 Unbalanced signal: US
3.3 Sporadic signal: SS
3.4 Parametric synthetic microbial datasets (varying signal branches)

4 Supplementary Note 2: Details about *treeclimbR*
4.1 The leaf FDR and candidates
4.2 The range of t
4.3 The selection of P-values is unbiased in branches without signals
4.4 An example on toy data

5 Supplementary Note 3: minP

Supplementary References

Supplementary Figures

1.1 Schematic overview of treeclimbR on the DS case

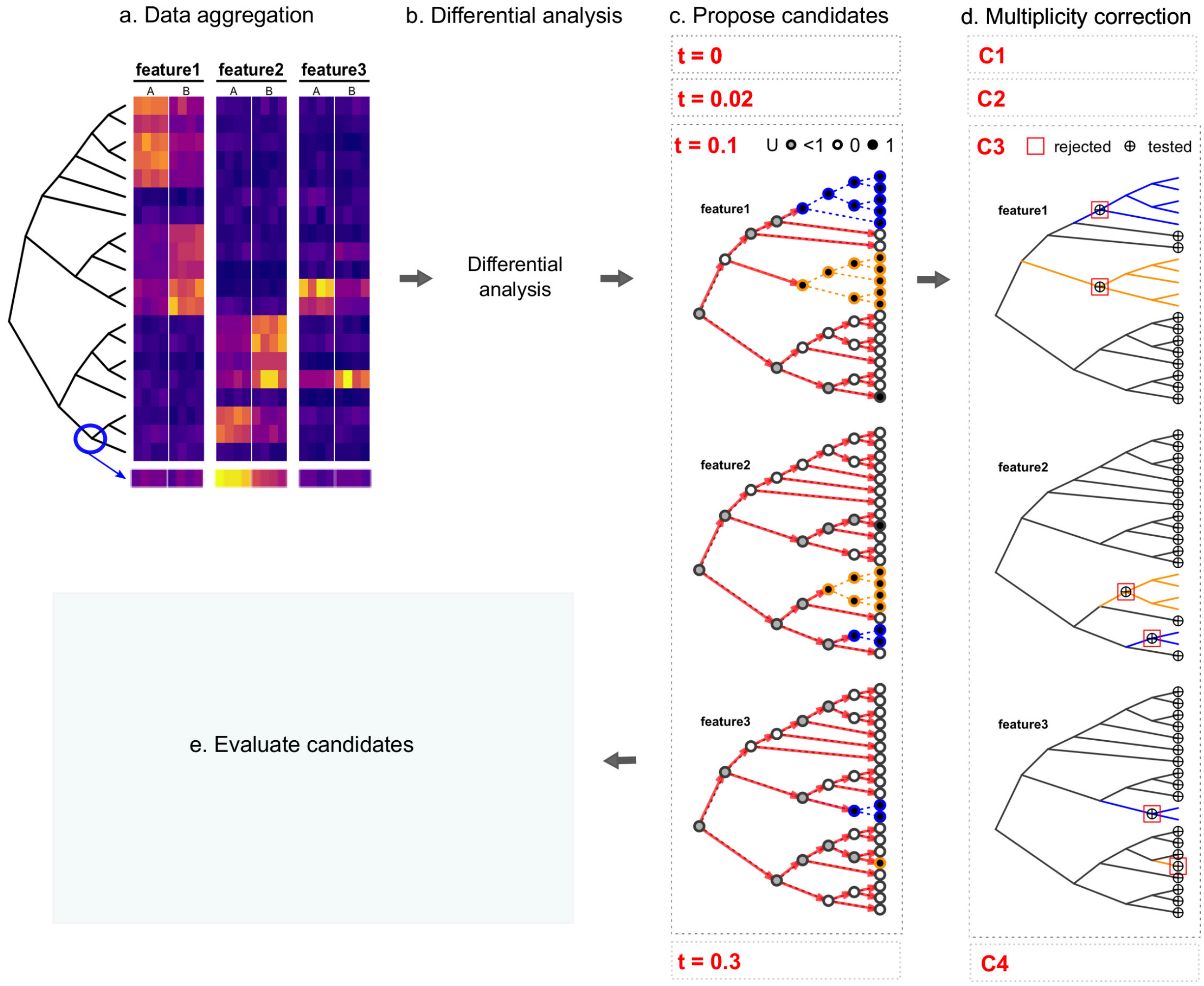

Figure S1. Schematic overview of the *treeclimbR* algorithm on the DS case. The overall procedure of *treeclimbR* is similar for the DS and the DA case (Figure 1). **(a)** Data aggregation. Each entity has multiple features (e.g., *feature1*, *feature2*, and *feature3*). Measurements of features on entities from samples across multiple groups (e.g., A and B) are shown in heatmaps. Different features have different signal patterns on the tree. **(b)** Differential analysis is run to estimate directions of changes and obtain P-values for features on each node. **(c)** Candidates are proposed at different t values. $t = 0.1$ is shown as an example. For each feature, the climbing starts from the root and stops when reaching leaves or nodes with $U = 1$. The global candidate at $t = 0.1$ includes sub-candidates of all features as shown as candidate $C3$ in **(d)**. Multiplicity correction in **(d)**, and the evaluation of candidates in **(e)** are similar to those of the DA case (Figure 1).

1.2 Results of parametric synthetic microbial datasets (fixed signal branches)

As shown in Fig. S2, nodes identified by *lasso*, *miLineage* are nested, where *miLineage1* and *miLineage2* are the one-part and two-part analyses provided by *miLineage*.

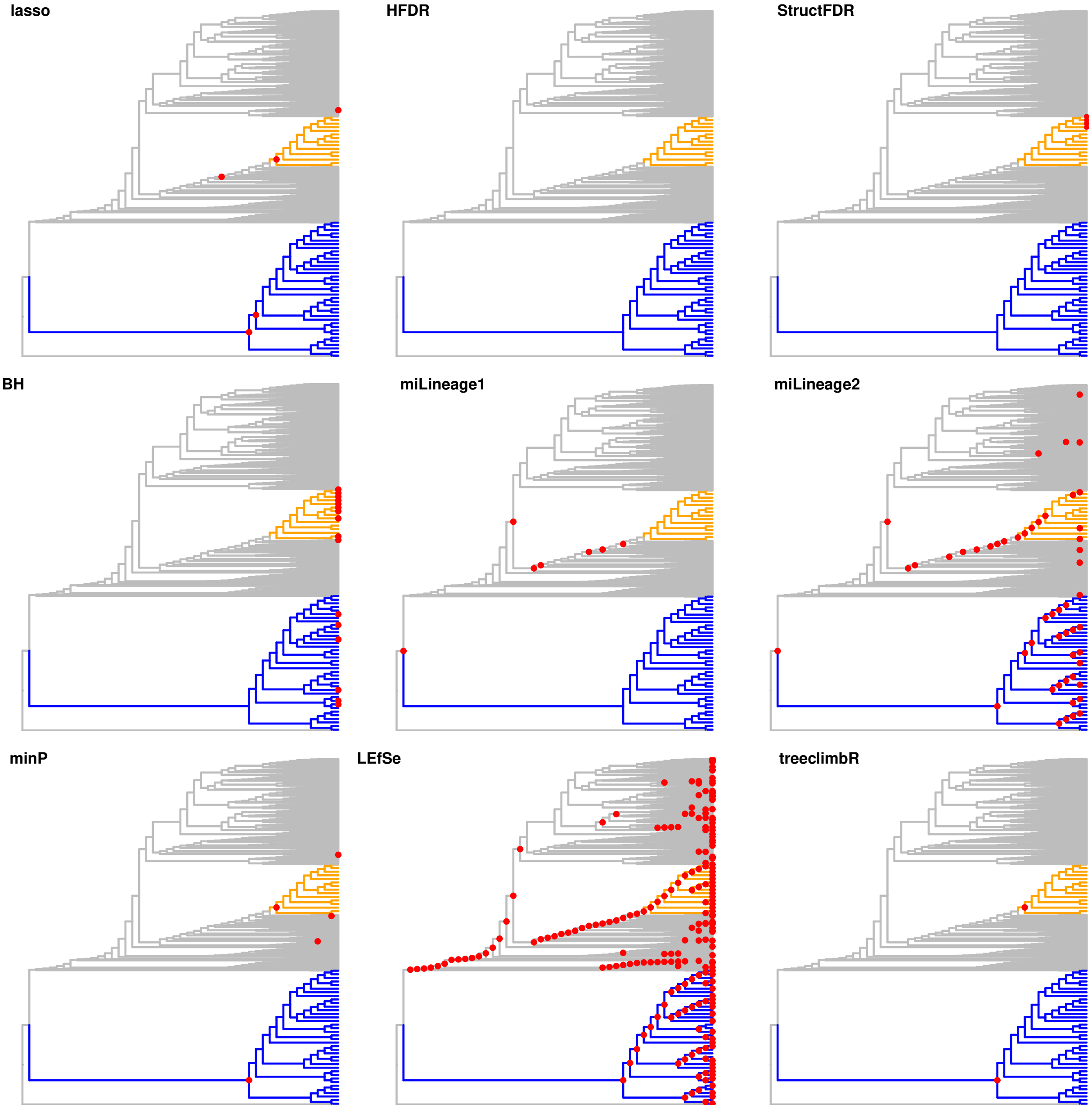

Figure S2. Results of methods in one repetition of BS with 25 samples per group. Two signal branches are colored in blue and orange. Nodes identified by methods are labeled as red points in each panel. *LEfSe*, *lasso* and *miLineage* (*miLineage1* and *miLineage2*) identify nodes that have ancestor-descendant relationship.

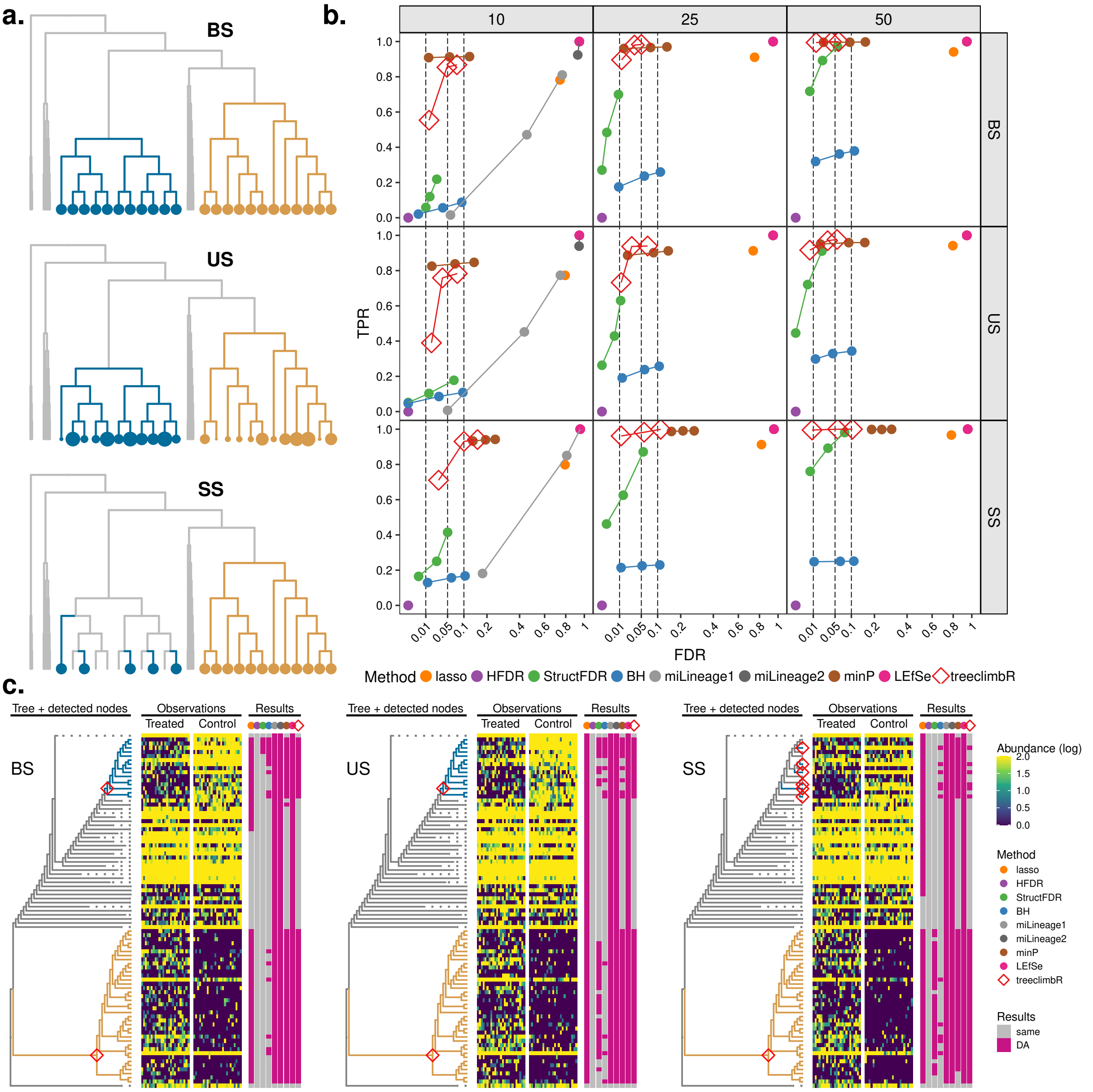

Figure S3. The performance of methods on parametric simulated microbial data. (a). A schematic example of three simulated scenarios: *BS*, *US* and *SS*. Signal branches (i.e., with DA between groups) are in turquoise (decreased) and gold (increased), and larger points represent bigger change. **(b).** Average TPR and FDR (over 100 repetitions) of methods on three scenarios across different sample sizes (10, 25, and 50 per group). Methods are in colors. Each method has three points that represent imposed FDR cutoffs at 0.01, 0.05 and 0.1. **(c).** DA branches identified by methods in one of 100 repetitions. The non-DA branches of the phylogenetic tree are represented with dashed lines to save space. Nodes identified by *treeclimbR* are labeled on the tree; and other methods are in Fig. S2. OTU counts are in heatmap with samples split by groups. All OTUs (rows of heatmap) identified by methods are summarized in the results panel.

Fig. S3 is similar to Figure 2, except that results of methods (*LEfSe*, *lasso* and *miLineage*) providing nested nodes are interpreted on broad resolution levels. Figure 2 uses identified nodes that have no descendant nodes identified; Supplementary Fig. S3 uses identified nodes with their ancestors not identified. For example, in the lasso panel of Fig. S2, two nodes (a parent node and a child node) are identified in the blue branch, the child is used in Figure 2 and the parent is used in Fig. S3. Therefore, in Fig. S3*b*, *lasso* has higher TPR but also much higher FDR than as shown in Figure 2. For *miLineage*, it has TPR equal to 1, but also

FDR equal to 1 in most simulations. In other words, *miLineage* overtakes signal branches and most identified branches are false positives. This could also be seen in the *miLineage1* and *miLineage2* panels of Fig. S2.

1.3 Results of non-parametric synthetic microbial datasets

Bichat *et al.* [1] have performed non-parametric simulations based on a real gut dataset from Brito *et al.* [2]. They first assigned 112 samples randomly to two groups, selected m taxa randomly from the most prevalent taxa, and multiplied counts of the m taxa in one of the two groups by a fold change fc. In other words, the tree is uninformative in the simulation. Different values of fc and m have been implemented. Bichat *et al.* have compared performances of the standard BH procedure and the tree-based method (*StructFDR*) using four different trees: taxonomic tree, random taxonomic tree, correlation tree and random correlation tree. Here, the taxonomic tree stores taxonomy information, and is independent of the abundance data that was used to perform differential analysis between groups. The random taxonomic tree was obtained by shuffling leaf labels of the taxonomic tree. The correlation tree was constructed based on correlations of taxa's abundance profiles. The random correlation tree was obtained by shuffling leaf labels of the correlation tree.

Results are shown in Figure 5 of Bichat *et al.* [1], highlighting that a tree-based method, *StructFDR*, has no gain and even performs worse (lower power) than the classical procedure *BH* when using the non-informative tree (taxonomic tree). To test whether our tree-based algorithm *treeclimbR* has the same issue, we reproduced their work and added *treeclimbR* to the comparison using the taxonomic tree. Results are in Fig. S4, which shows that *treeclimbR* performs similarly to *BH* when the tree is uninformative. We show results of *treeclimbR* based on correlation trees in Fig. S5 to understand the performance of *treeclimbR* in the case of double-dipping (i.e., using the same data to construct the tree and to perform differential abundance analysis).

We again follow the work of Bichat *et al.* [1] as described above to simulate abundance data of taxa for two groups, and further construct correlation trees based on the simulated abundance data. More precisely, the correlation tree is constructed by first computing the pairwise Spearman correlations of taxa with shared zero counts in samples excluded. Then, the correlation matrix ($\mathbf{C}$) is converted into a dissimilarity matrix ($\mathbf{D} = \mathbf{1} - \mathbf{C}$), on which the tree is built using hierarchical clustering with Ward linkage. Here, we construct correlation trees using different samples: tree *cor1* using samples in one group; tree *cor2* using samples in the other group; and tree *cor* using samples from both groups. To identify differential abundance taxa, we perform Wilcoxon rank sum tests to obtain P-values and adjust for multiple hypotheses using *BH*, *StructFDR* and *treeclimbR*; results based on 100 repetitions are shown in Fig. S5. We see that *treeclimbR* based on tree *Cor1* or tree *Cor2* has similar results to *BH.* However, *treeclimbR* based on tree *Cor* has higher TPR and also inflated *FDR* compared to *BH.* This is due to the double-use of the data: first to create the correlation tree, and then second to perform differential abundance test. When using all samples to construct tree *Cor*, we incorporate not only the similarity of taxa's abundance profiles but also the information about group differences in the tree construction.

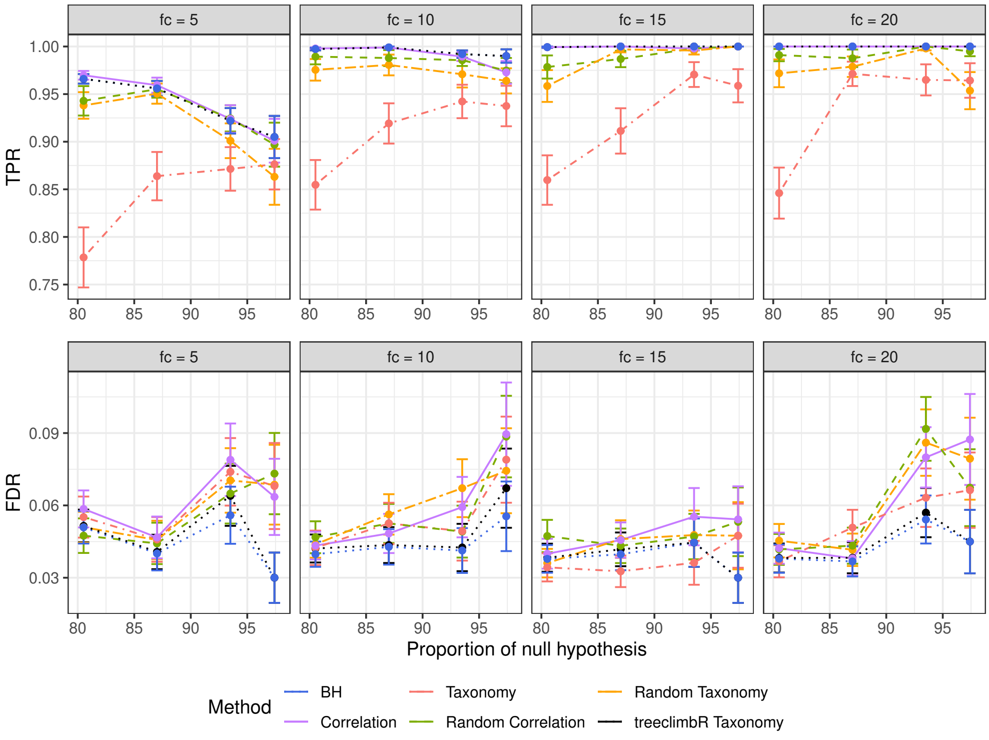

Figure S4. Results of different FDR control procedures on non-parametric simulation. Mean and Squared Error of the Mean (SEM) of the true positive rates (TPR, top) and FDR (bottom) per different fold changes (facets) for non-parametric simulations. The different FDR control procedures, including standard Benjamini-Hochberg (*BH*), *StructFDR* based on different trees (*Taxonomy, Random Taxonomy, Correlation, Random Correlation*) and *treeclimbR* based on taxonomy tree, are color-coded. Mean and SEM are computed over 100 replicates.

1.4 Results of BCR-XL-sim datasets

In addition to *pS6*, protein markers including *pPIcg2*, *pErk*, *pNFkB* are also differentially expressed in B cells when comparing stimulated samples against control samples. In Fig. S6, we see that only *treeclimbR* identifies the big branch of B cells for three markers; other methods either find some sub-branches or miss the whole big branch of B cells. By scanning the whole tree to find the suitable resolution, *treeclimbR* could perform well on high-resolution clusters (e.g. 2500 clusters) that becomes difficult for *diffcyt* (See Fig. S6d).

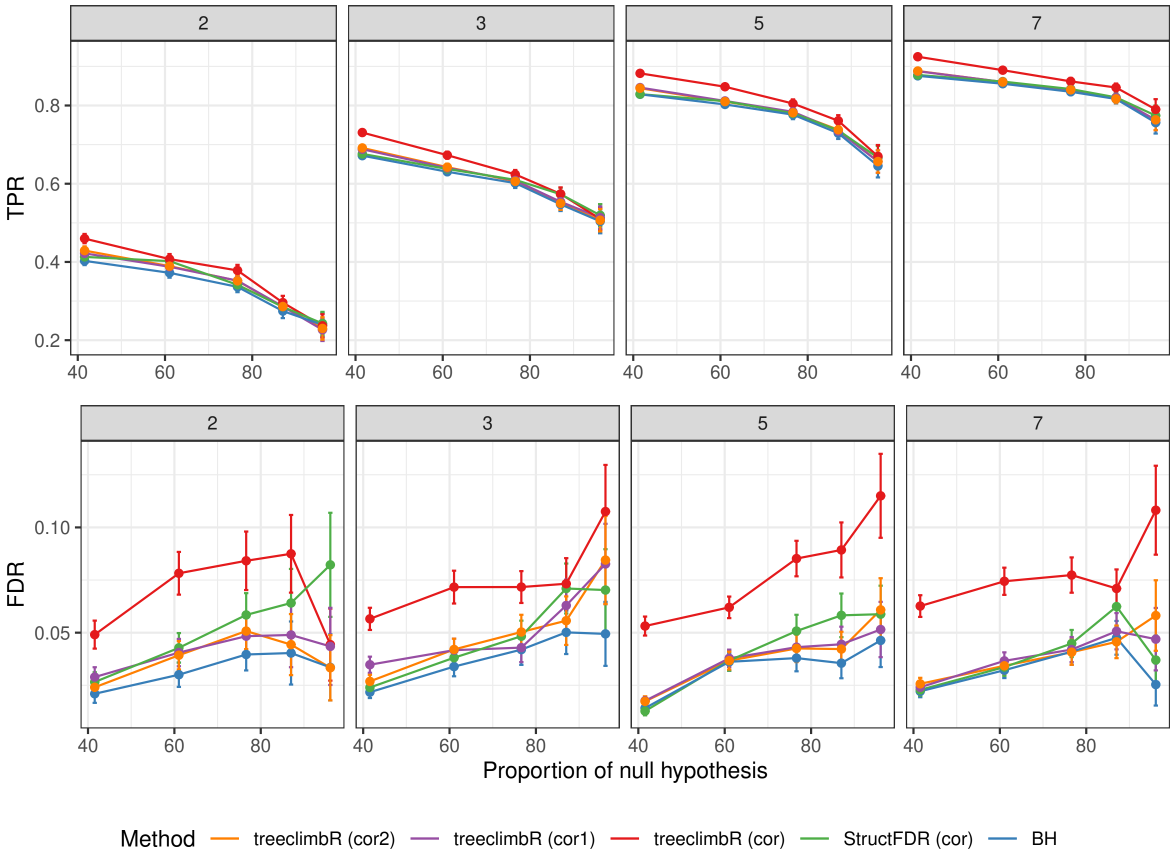

Figure S5. Results of different FDR control procedures on non-parametric simulation. Mean and Squared Error of the Mean (SEM) of the true positive rates (TPR, top) and FDR (bottom) per different fold changes (facets) for non-parametric simulations. Methods include tree-free method (*BH*), *StructFDR* based on tree *Cor*, and *treeclimbR* based on trees (*Cor*, *Cor1*, *Cor2*). Mean and SEM are computed over 100 replicates.

1.5 Benchmark methods' runtimes

We benchmark the run time of methods for data across different sizes. Here, the size of data is varied by changing the number of entities (100, 500, 1000, 3000, 6000, and $10,000$), and the total number of samples (100 and 500). Counts of entities for each sample are drawn from a multinomial distribution:

$$\textbf{Mult}(n, p)$$

where the total count n is the number of entities multiplied by 20, and p is randomly generated from a uniform distribution and further normalized to sum to 1. Samples are randomly assigned into two groups (control and treated). For those in the treated group, the first 30% entities are selected and their counts are multiplied by 8 to simulate differences between two groups. Tree structures of entities are randomly generated with *rtree* from R package *ape*. The benchmark is performed in one single processor core using the following softwares:

- *StructFDR*: *StructFDR (v1.3)*
- *miLineage*: *miLineage (v2.1)*
- *treeclimbR*: *treeclimbR*
- *lasso*: *glmnet (v2.0-18)*

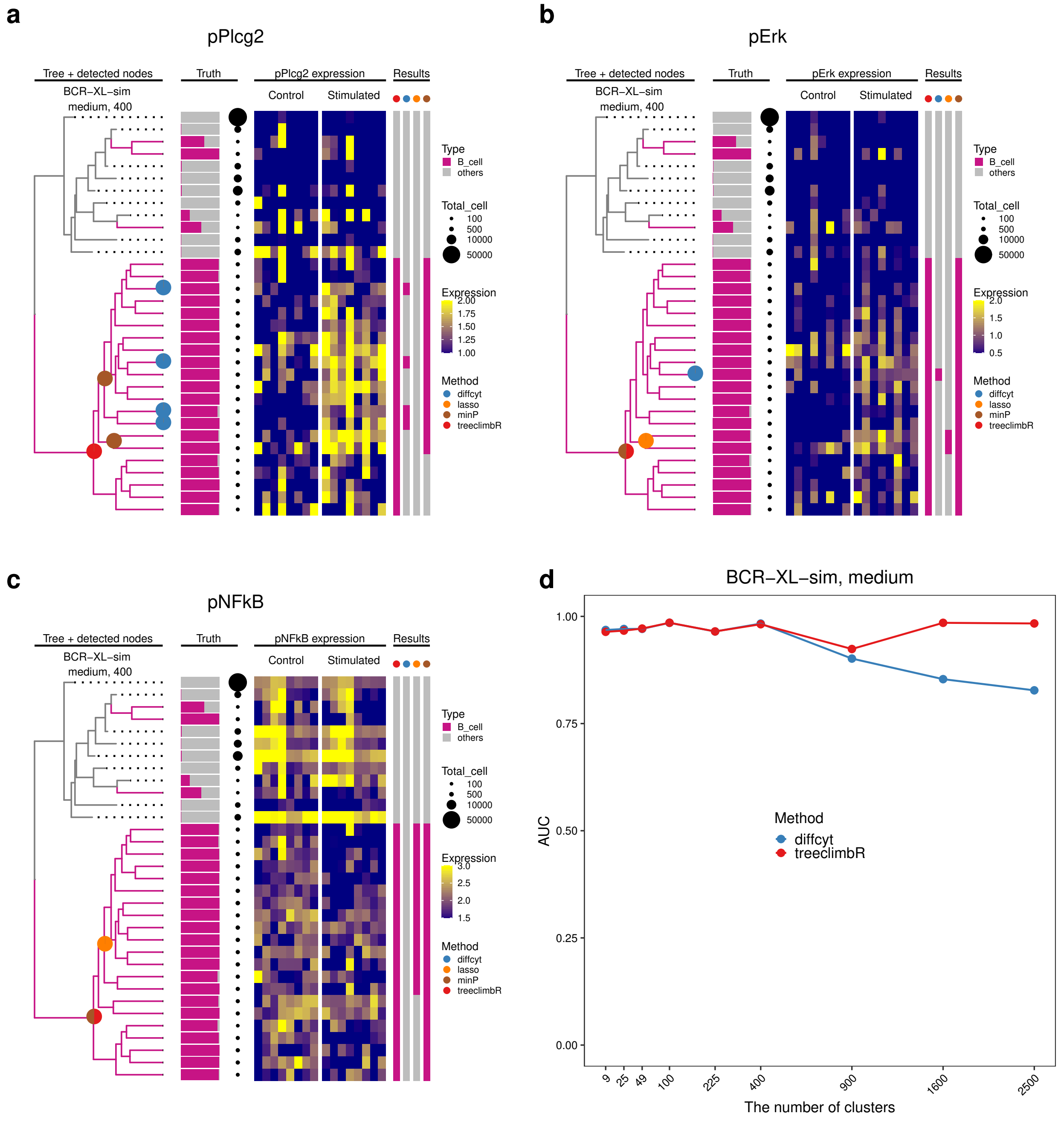

Figure S6. Results of methods on the medium scenario of *BCR-XL-sim*. Branches that are identified to have differential expression of *pPIcg2*, *pErk* and *pNFkB* in the medium scenario of *BCR-XL-sim* are shown correspondingly in plots **a, b, c**. Each plot has four panels showing tree, truth, observations and results. Non-DS branches (without B cells) are represented in dashed lines to save space; DS branches (with B cells above 50%) are in purple. Nodes identified by methods are in different colors. The true cell-type compositions and cell counts (point sizes) on each leaf are given in the truth panel. The median expressions of marker on leaves are shown as heatmap with cell subpopulations in rows and samples in columns (split by groups). Descendant leaves of identified nodes are annotated in the results panel. **d** Area under ROC curves (AUC) of *diffcyt* and *treeclimbR* when using different numbers of clusters (between 9 and 2500 clusters).

- *BH*: *stats*
- *HFDR*: *structSSI (v1.1.1)*
- *minP*: *treeclimbR*
- *LEfSe*: *lefse* in Conda with *python 2.7*

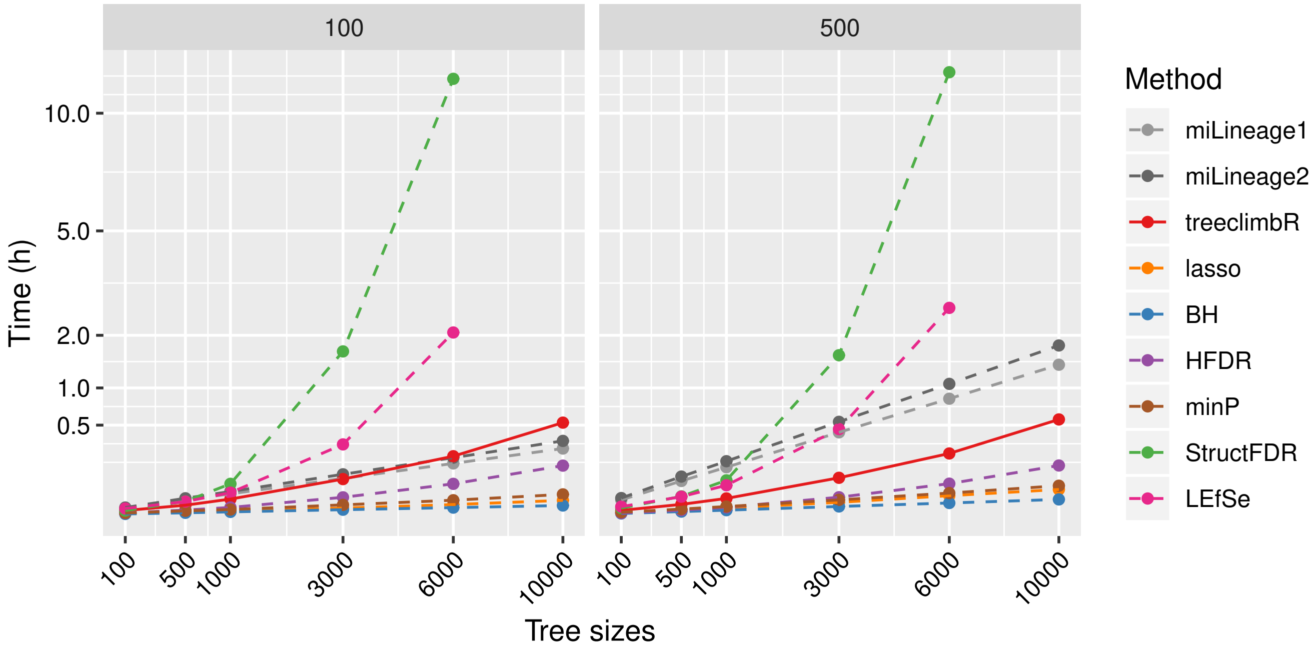

Figure S7. Benchmark of method run times for different tree sizes using a single processor core. Methods are in colors. Run times (in hours) of methods increase as tree sizes (the number of leaves) get larger. Run times of the same method are connected with dashed lines for all methods except *treeclimbR*. Methods are in colors. Two methods, *StructFDR* and *LEfSe* have missing results for the tree with $10,000$ leaves. The former took more than 24 hours and was stopped before finish; the latter stopped automatically with errors related to the long run time.

Results of the run time benchmark are shown in Fig. S7 under $FDR = 0.05$. Note that some tree-based methods (e.g., *miLineage* and *LEFse*) do not accept the tree format directly as the input, which requires additional time in data preparation. To show better performance of competitors, we do not include the time of data preparation in the run time for methods other than *treeclimbR* and *minP*. Also, to obtain results for different FDR levels, tree-based methods (*miLineage*, *HFDR*, *StructFDR*) would need to rerun the whole procedure every time. However, *treeclimbR* that separates the candidate proposal step and the multiplicity correction step, only need to find candidates (the most computation intensive step) once, and use them to correct FDR at different levels.

Supplementary Tables

2.1 Results of miRNA data

Region	miRNAs
chr1:195037040-195037634	miRNA-29C, miRNA-29b-2
chr1:162217814-162223477	miRNA-199a-2, miRNA-214
chr11:70234717-70235135	miRNA-195a, miRNA-497a
chr12:109563692-109595388	miRNA-127, miRNA-136, miRNA-337, miRNA-431, miRNA-433, miRNA-434, miRNA-540, miRNA-673
chr12:109709060-109747960	miRNA-134, miRNA-154, miRNA-299a, miRNA-300, miRNA-329, miRNA-369, miRNA-376, miRNA-377, miRNA-379, miRNA-380, miRNA-381, miRNA-382, miRNA-409, miRNA-410, miRNA-411, miRNA-485, miRNA-541, miRNA-667, miRNA-668
chr9:51103034-51103645	miRNA-34c, miRNA-34b
chrX:19146294-19146971	miRNA-221, miRNA-222
chrX:7237683-7248497	miRNA-188, miRNA-362, miRNA-500, miRNA-501, miRNA-532

Table S 1. miRNAs in 8 identified genomic clusters.

2.2 Results of parametric synthetic microbial datasets (fixed signal branches)

		BS						US						SS					
		TPR			FDR			TPR			FDR			TPR			FDR		
alpha	method	10	25	50	10	25	50	10	25	50	10	25	50	10	25	50	10	25	50
**0.01**	BH	0.021	0.175	0.319	0.003	0.009	0.013	0.046	0.191	0.298	0.000	0.013	0.013	0.130	0.214	0.248	0.012	0.012	0.011
	HFDR	0.000	0.000	0.000	0.000	0.000	0.000	0.000	0.000	0.000	0.000	0.000	0.000	0.000	0.000	0.000	0.000	0.000	0.000
	lasso	0.296	0.321	0.399	0.366	0.385	0.313	0.320	0.372	0.339	0.404	0.329	0.340	0.332	0.361	0.520	0.369	0.334	0.194
	LEfSe	0.940	1.000	1.000	0.379	0.425	0.481	0.951	0.996	1.000	0.357	0.411	0.478	0.988	1.000	1.000	0.461	0.503	0.514
	miLineage1	0.006	0.298	0.283	0.057	0.718	0.341	0.007	0.265	0.165	0.050	0.512	0.005	0.035	0.118	0.036	0.049	0.799	0.562
	miLineage2	0.146	0.451	0.919	0.859	0.241	0.034	0.168	0.444	0.956	0.848	0.220	0.034	0.231	0.893	0.999	0.824	0.257	0.213
	minP	0.909	0.960	0.996	0.013	0.016	0.025	0.825	0.887	0.951	0.018	0.021	0.020	0.933	0.987	0.998	0.135	0.156	0.186
	StructFDR	0.058	0.270	0.718	0.010	0.000	0.006	0.051	0.263	0.446	0.000	0.000	0.000	0.165	0.462	0.760	0.003	0.001	0.007
	treeclimbR	0.553	0.896	0.994	0.014	0.012	0.013	0.390	0.732	0.916	0.017	0.012	0.006	0.710	0.962	0.995	0.030	0.012	0.009
**0.05**	BH	0.056	0.236	0.362	0.039	0.058	0.062	0.085	0.238	0.329	0.030	0.059	0.044	0.157	0.225	0.250	0.060	0.052	0.064
	HFDR	0.000	0.000	0.000	0.000	0.000	0.000	0.000	0.000	0.000	0.000	0.000	0.000	0.000	0.000	0.000	0.000	0.000	0.000
	lasso	0.296	0.321	0.399	0.366	0.385	0.313	0.320	0.372	0.339	0.404	0.329	0.340	0.332	0.361	0.520	0.369	0.334	0.194
	LEfSe	0.986	1.000	1.000	0.853	0.831	0.869	0.986	0.999	1.000	0.842	0.832	0.851	0.998	1.000	1.000	0.867	0.872	0.861
	miLineage1	0.277	0.280	0.280	0.367	0.633	0.300	0.290	0.208	0.169	0.344	0.231	0.108	0.412	0.072	0.077	0.481	0.615	0.588
	miLineage2	0.208	0.638	0.972	0.827	0.267	0.246	0.235	0.623	0.990	0.814	0.279	0.180	0.315	0.980	1.000	0.785	0.349	0.321
	minP	0.913	0.966	0.996	0.055	0.076	0.094	0.838	0.902	0.958	0.071	0.086	0.091	0.940	0.991	0.998	0.193	0.212	0.239
	StructFDR	0.120	0.484	0.892	0.015	0.001	0.023	0.103	0.429	0.721	0.014	0.005	0.005	0.250	0.625	0.892	0.026	0.015	0.034
	treeclimbR	0.854	0.980	0.999	0.048	0.034	0.041	0.759	0.937	0.970	0.038	0.029	0.033	0.930	0.983	1.000	0.100	0.057	0.057
**0.10**	BH	0.088	0.260	0.379	0.092	0.110	0.111	0.108	0.257	0.343	0.096	0.106	0.101	0.168	0.230	0.251	0.104	0.108	0.109
	HFDR	0.000	0.000	0.000	0.000	0.000	0.000	0.000	0.000	0.000	0.000	0.000	0.000	0.000	0.000	0.000	0.000	0.000	0.000
	lasso	0.296	0.321	0.399	0.366	0.385	0.313	0.320	0.372	0.339	0.404	0.329	0.340	0.332	0.361	0.520	0.369	0.334	0.194
	LEfSe	0.993	1.000	1.000	0.929	0.911	0.925	0.996	1.000	1.000	0.924	0.922	0.927	1.000	1.000	1.000	0.931	0.931	0.931
	miLineage1	0.560	0.276	0.287	0.661	0.566	0.339	0.564	0.193	0.182	0.635	0.174	0.170	0.525	0.049	0.099	0.664	0.587	0.636
	miLineage2	0.249	0.768	0.986	0.811	0.381	0.392	0.264	0.743	0.999	0.788	0.357	0.346	0.406	0.989	1.000	0.755	0.468	0.460
	minP	0.915	0.970	0.998	0.121	0.136	0.156	0.847	0.912	0.959	0.140	0.142	0.154	0.942	0.991	0.999	0.244	0.274	0.297
	StructFDR	0.218	0.700	0.972	0.026	0.009	0.056	0.178	0.630	0.911	0.067	0.011	0.022	0.415	0.871	0.981	0.050	0.055	0.077
	treeclimbR	0.868	0.983	0.999	0.076	0.050	0.059	0.783	0.939	0.977	0.078	0.067	0.055	0.941	0.999	1.000	0.155	0.111	0.104

Table S 2. Average TPR and FDR (over 100 repetitions) of methods on parametric simulated microbial data. Simulated data includes three scenarios across different sample sizes (10, 25, and 50) per group. The average TPR and FDR are calculated at three imposed FDR cutoffs (0.01, 0.05 and 0.1). This is a tabular form of Figure 2b.

2.3 Results of AML-sim datasets

		Weak						Medium						Strong					
		TPR			FDR			TPR			FDR			TPR			FDR		
alpha	method	400	900	1600	400	900	1600	400	900	1600	400	900	1600	400	900	1600	400	900	1600
**0.01**	diffcyt	0	0	0	0	0	0	0.89	0.875	0.717	0.006	0.017	0.003	0.971	0.950	0.965	0.058	0.084	0.023
	HFDR	0	0	0	0	0	0	0.00	0.000	0.000	0.000	0.000	0.000	0.000	0.000	0.000	0.000	0.000	0.000
	lasso	0	0	0	0	0	0	0.89	0.882	0.956	0.006	0.031	0.085	0.935	0.964	0.960	0.002	0.032	0.009
	minP	0	0	0	0	0	0	0.89	0.875	0.901	0.006	0.017	0.008	0.952	0.964	0.960	0.057	0.300	0.009
	StructFDR	0	0	0	0	0	0	0.89	0.835	0.717	0.006	0.005	0.003	0.935	0.893	0.955	0.002	0.003	0.008
	treeclimbR	0	0	0	0	0	0	0.89	0.875	0.901	0.006	0.017	0.008	0.935	0.964	0.960	0.002	0.032	0.009
**0.05**	diffcyt	0	0	0	1	0	0	0.89	0.875	0.717	0.006	0.017	0.003	0.971	0.950	0.965	0.161	0.131	0.023
	HFDR	0	0	0	0	0	0	0.00	0.000	0.000	0.000	0.000	0.000	0.000	0.000	0.000	0.000	0.000	0.000
	lasso	0	0	0	0	0	0	0.89	0.882	0.956	0.006	0.031	0.085	0.935	0.964	0.960	0.002	0.032	0.009
	minP	0	0	0	1	0	0	0.89	0.875	0.901	0.006	0.017	0.008	0.952	0.964	0.970	0.057	0.497	0.123
	StructFDR	0	0	0	0	0	0	0.89	0.835	0.717	0.006	0.005	0.003	0.935	0.926	0.962	0.002	0.004	0.009
	treeclimbR	0	0	0	1	0	0	0.89	0.875	0.901	0.006	0.017	0.008	0.952	0.964	0.960	0.057	0.032	0.009
**0.1**	diffcyt	0	0	0	1	0	0	0.89	0.875	0.717	0.006	0.017	0.003	0.971	0.950	0.965	0.161	0.156	0.023
	HFDR	0	0	0	0	0	0	0.00	0.000	0.000	0.000	0.000	0.000	0.000	0.000	0.000	0.000	0.000	0.000
	lasso	0	0	0	0	0	0	0.89	0.882	0.956	0.006	0.031	0.085	0.935	0.964	0.960	0.002	0.032	0.009
	minP	0	0	0	1	0	0	0.89	0.875	0.901	0.006	0.017	0.008	0.959	0.964	0.970	0.172	0.548	0.123
	StructFDR	0	0	0	0	0	0	0.89	0.835	0.717	0.006	0.005	0.003	0.953	0.935	0.962	0.006	0.004	0.009
	treeclimbR	0	0	0	1	0	0	0.89	0.875	0.901	0.006	0.017	0.008	0.952	0.964	0.965	0.057	0.083	0.123

Table S 3. TPR and FDR of methods on *AML-sim* data. Simulated data includes three scenarios (weak, medium, and strong). Trees are constructed at three different sizes (400, 900, and 1600 leaves). TPR and FDR are computed at three imposed FDR cutoffs (0.01, 0.05 and 0.1). This is a tabular form of Figure 3a.

Supplementary Note 1: Simulation framework

In Fig. S8, the height of the bar represents the proportion of entities (in the specified branch) within a sample. We consider the tree structure as three parts, the two selected DA-branches and the rest. The phenotypic outcome is represented by the group (control or treatment). The proportions of the orange and blue branches are swapped between different groups in scenario *BS* and *US*. The height of the orange bar in the treatment group is equal to the blue bar in the control group, and the height of the blue bar in the treatment group is the same as that of the orange bar in the control group. In *BS*, the increase or decrease in the proportion is evenly distributed to the entities within branch by a common factor r (in the blue branch) or $\frac{1}{r}$ (in the orange branch). However, in *US*, the change of proportion is not evenly distributed and the factor r_k varies on entities. In *SS*, the swap is partial and depends on the value c. That's why the height of the orange bar in the treatment group is different to that of the blue bar in the control group.

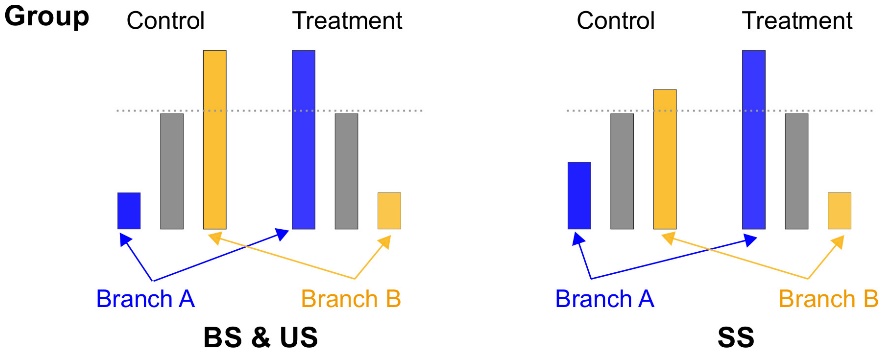

Figure S8. The overall idea of simulated scenarios.

3.1 Balanced signal: BS

Proof: The proportions of two branches are swapped between groups in BS.

$$\begin{aligned}
\sum_{g\in A}\hat{\pi}_g^T &= \sum_{g\in A} r_g\hat{\pi}_g^C = \sum_{g\in A}\hat{\pi}_g^C r_g \\
&= \sum_{g\in A}\hat{\pi}_g^C\left(1+\frac{\sum_{k\in B}\hat{\pi}_k^C-\sum_{g\in A}\hat{\pi}_g^C}{\sum_{g\in A}\hat{\pi}_g^C u_g}u_g\right) \\
&= \sum_{g\in A}\hat{\pi}_g^C+\sum_{g\in A}\hat{\pi}_g^C\frac{\sum_{k\in B}\hat{\pi}_k^C-\sum_{g\in A}\hat{\pi}_g^C}{\sum_{g\in A}\hat{\pi}_g^C u_g}u_g \\
&= \sum_{g\in A}\hat{\pi}_g^C+\left(\sum_{k\in B}\hat{\pi}_k^C-\sum_{g\in A}\hat{\pi}_g^C\right)\frac{\sum_{g\in A}\hat{\pi}_g^C u_g}{\sum_{g\in A}\hat{\pi}_g^C u_g} \\
&= \sum_{k\in B}\hat{\pi}_k^C
\end{aligned} \tag{1}$$

Similarly, we could have $\sum_{k\in B}\hat{\pi}_k^T = \sum_{g\in A}\hat{\pi}_g^C$.

3.2 Unbalanced signal: US

Unbalanced signal (US). Two branches (A and B)are randomly selected to swap their proportion.

$$\begin{cases} \hat{\pi}_k^T = \hat{\pi}_k^C; & k \notin A, B \\ \hat{\pi}_k^T = r_k \hat{\pi}_k^C, \quad r_k = 1 + \frac{\sum_{k\in B} \hat{\pi}_k^C - \sum_{k\in A} \hat{\pi}_k^C}{\sum_{k\in A} \hat{\pi}_k^C u_k} u_k; & k \in A \\ \hat{\pi}_k^T = r_k \hat{\pi}_k^C, \quad r_k = 1 + \frac{\sum_{k\in A} \hat{\pi}_k^C - \sum_{k\in B} \hat{\pi}_k^C}{\sum_{k\in B} \hat{\pi}_k^C u_k} u_k & k \in B \end{cases} \tag{2}$$

where u_k is randomly generated for entity k from a uniformly distributed function as Equation 3 and b is set to make sure r_k is above zero.

$$\begin{cases} u_k \sim \mathrm{U}(b, 1), \quad b = \max(1 - \frac{\sum_{k\in B} \hat{\pi}_k^C}{\sum_{k\in A} \hat{\pi}_k^C}, 0); & k \in A \\ u_k \sim \mathrm{U}(b, 1), \quad b = \max(1 - \frac{\sum_{k\in A} \hat{\pi}_k^C}{\sum_{k\in B} \hat{\pi}_k^C}, 0); & k \in B \end{cases} \tag{3}$$

The proof about the proportions of two branches being swapped is similar to Equation 1.

3.3 Sporadic signal: SS

Sporadic signal (SS). Two branches (A and B)are randomly selected, but only some leaves in branch A (denoted as A') are randomly selected to swap their proportion with leaves in branch B.

$$\begin{cases} \hat{\pi}_k^T = \hat{\pi}_k^C; & k \notin A', B \\ \hat{\pi}_k^T = r\hat{\pi}_k^C; & k \in A' \\ \hat{\pi}_k^T = c\hat{\pi}_k^C; & k \in B \end{cases} \tag{4}$$

where $r = \frac{(1-c)\sum_{k\in B} \hat{\pi}_k + \sum_{k\in A'} \hat{\pi}_k}{\sum_{k\in A'} \hat{\pi}_k}$ and $0 < c < 1$. The proof about the proportions of two branches being swapped is similar to Equation 1.

3.4 Parametric synthetic microbial datasets (varying signal branches)

We have further varied signal branches randomly to simulate data for three scenarios: BS, US, and SS. The simulation procedure is summarized as below.

- Step 1: we categorize nodes of the tree into ranges $[1, 5]$, $[6, 10]$, $[11, 15]$, $[16, 20]$, $[21, 25]$, $[26, 30]$, $[31, 35]$, $[36, 40]$, $[41, 45]$, $[46, 50]$ and above, according to their numbers of descendant leaves. For each of the listed 10 ranges, we randomly select 10 nodes to form a set A.

- Step 2: for each repetition of a scenario, two signal branches (a and b) are randomly selected to swap their proportion: a is randomly selected from the set A, and b from the tree. The two branches a and b cannot overlap. We keep the result if the ratio of branch b's proportion to branch a's proportion is in the range $[2, 9]$; otherwise, a new random draw is made.

- Step 3: we repeat step 2 until there are 100 repetitions for each of the three scenarios.

- Step 4: for each repetition in three scenarios, OTU counts across samples are generated from negative binomial distributions (see Methods section of the paper). We simulate two different sample sizes: 25 samples per group and 153 samples per group.

Step 1 is performed to have signals at varying branch sizes. Here, an internal node represents a branch that includes the internal node and all its descendant nodes. The tree used in the simulation is close to a binary tree that has each internal node with two child nodes. Such a tree naturally has many more internal nodes with fewer descendant leaves. Without step 1, the simulation is biased to have overwhelming small signal branches. In step 2, the range $[2, 9]$ is used to make sure there are reasonable differences simulated. In step 3, 153 is the number of samples in $throat\_v35$ that our simulation is based on.

We display in Fig. S9 that the sizes of signal branches vary among 100 repetitions. Some repetitions have two signal branches with very similar size; others have very different branch sizes. Fig. S10 shows the absolute log_2FC of leaves in signal branches over 100 repetitions for three simulated scenarios. Fig. S11 shows relative abundances of two signal branches for each of 100 repetitions in three simulated scenarios. Performance of methods are compared in Fig. S12. We further show variations of FDR and TPR of three top tree-based methods that are in the top left corner of Fig. S12 with the *BH* method in Fig. S13 and Fig. S14 for simulations with different sample sizes. In Fig. S12, *treeclimbR* has strong performance with high TPR and controlled FDR; *StructFDR* has improved TPR when compared to tree-free method *BH*; *minP* has high TPR but inflated FDR when the sample size is increased from 25 samples to 153 samples per group; *lasso* is slightly improved with larger sample size, but still has lower TPR and higher TPR compared to *StructFDR* and *treeclimbR*. In Fig. S13 and Fig. S14, variations across 100 repetitions for *BH*, *StructFDR* and *treeclimbR* are similar; *minP* has a larger variation of FDR under 153 samples per group; TPR of three tree-based methods (*minP*, *StructFDR*, *treeclimbR* are improved when compared to the *BH* method.

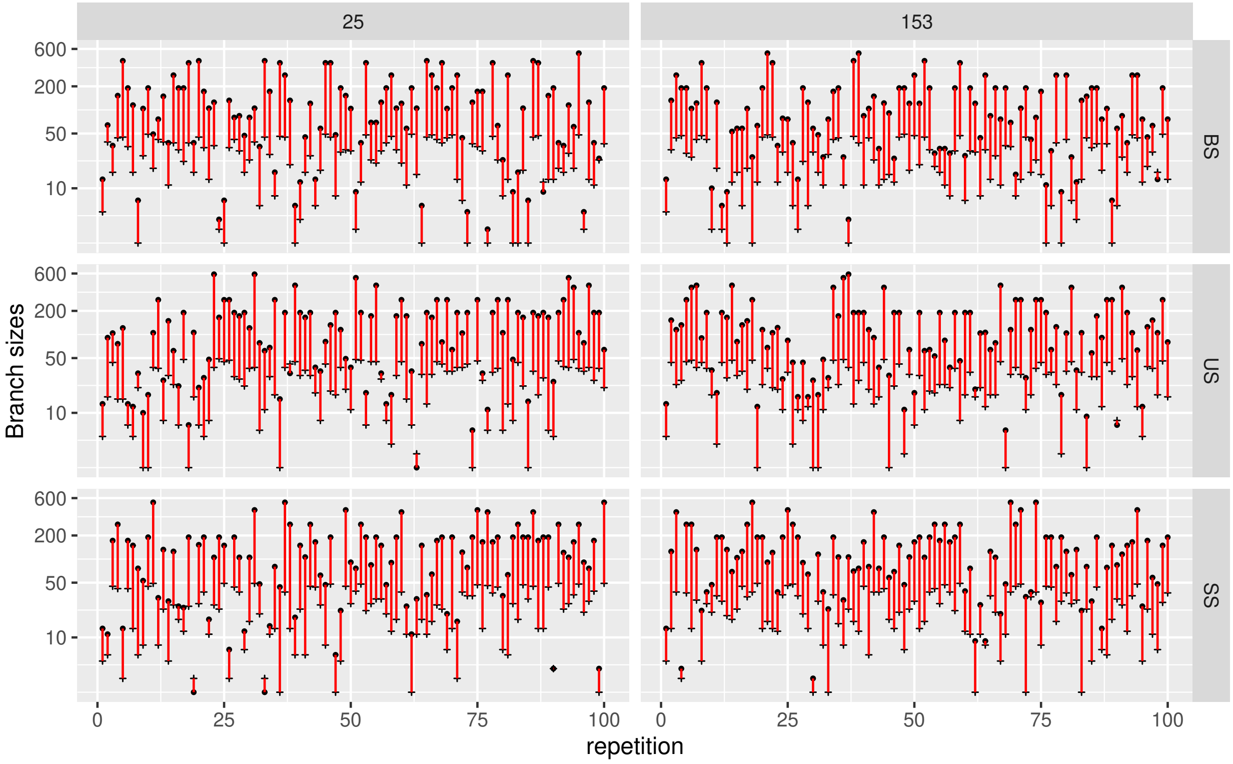

Figure S9. The sizes of signal branches in 100 repetitions for three simulated scenarios of parametric simulated microbial data. The left and the right panels are for simulation with 25 and 153 samples per group, respectively. Sizes of branches a and b generated in the simulation step 2 are shown as points with the shape $(+)$ and $(.)$, respectively.

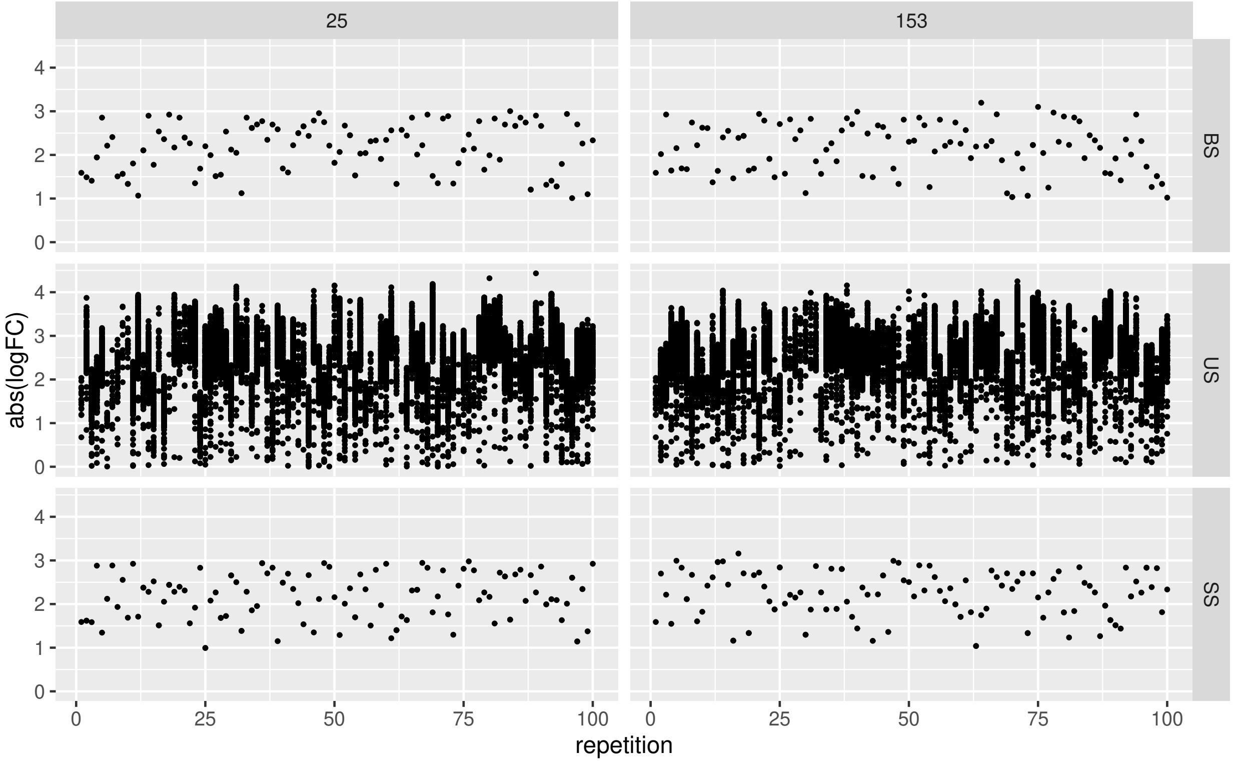

Figure S10. Absolute log_2FC of signal leaves on parametric simulated microbial data. Results on the left and the right panels are for simulation with 25 and 153 samples per group, respectively. Each panel has three plots for the three scenarios (BS, US, and SS) and 100 repetitions. Note: Signal leaves in BS or SS each have the same absolute log_2FC, hence they overlap as a single point.

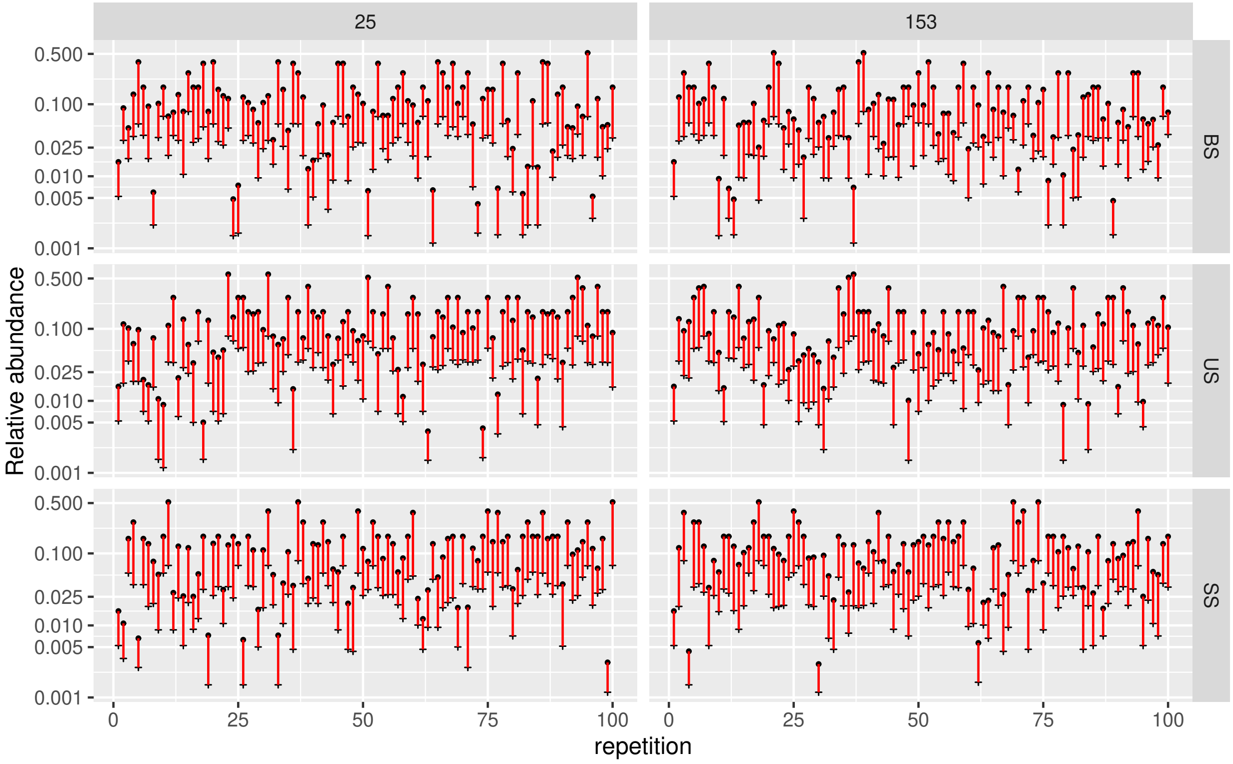

Figure S11. Relative abundance of two signal branches on parametric simulated microbial data. Results on the left and the right panels are for simulation with 25 and 153 samples per group, respectively. Each panel has three plots for the three scenarios (BS, US, and SS) and 100 repetitions.

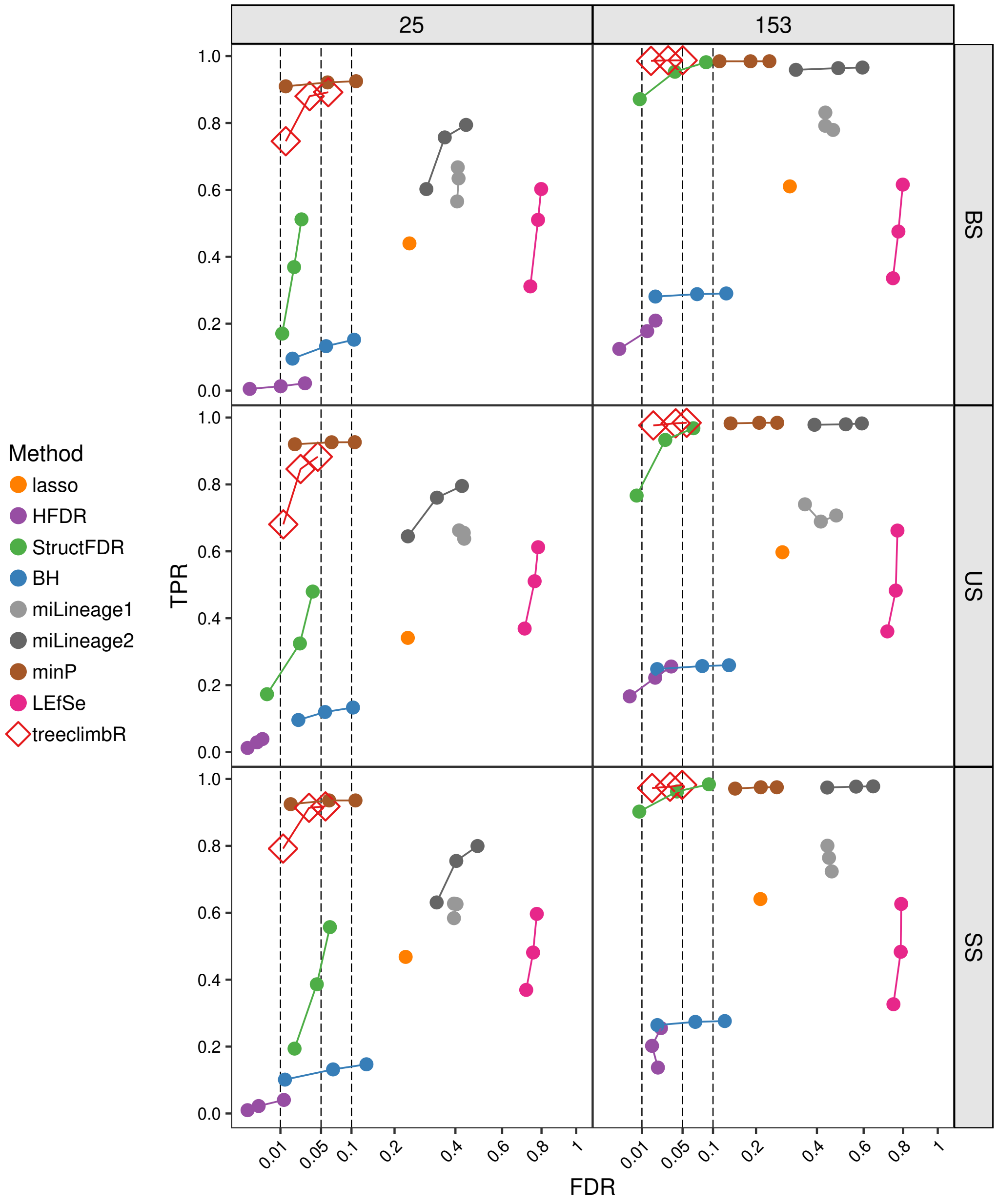

Figure S12. The performance of methods on parametric simulated microbial data. Average TPR and FDR (over 100 repetitions) of methods on three scenarios (BS, US, and SS) across different sample sizes (25 and 153 per group). Methods are in colors. Each method has three points that represent imposed FDR cutoffs at 0.01**,** 0.05**, and** 0.1**.**

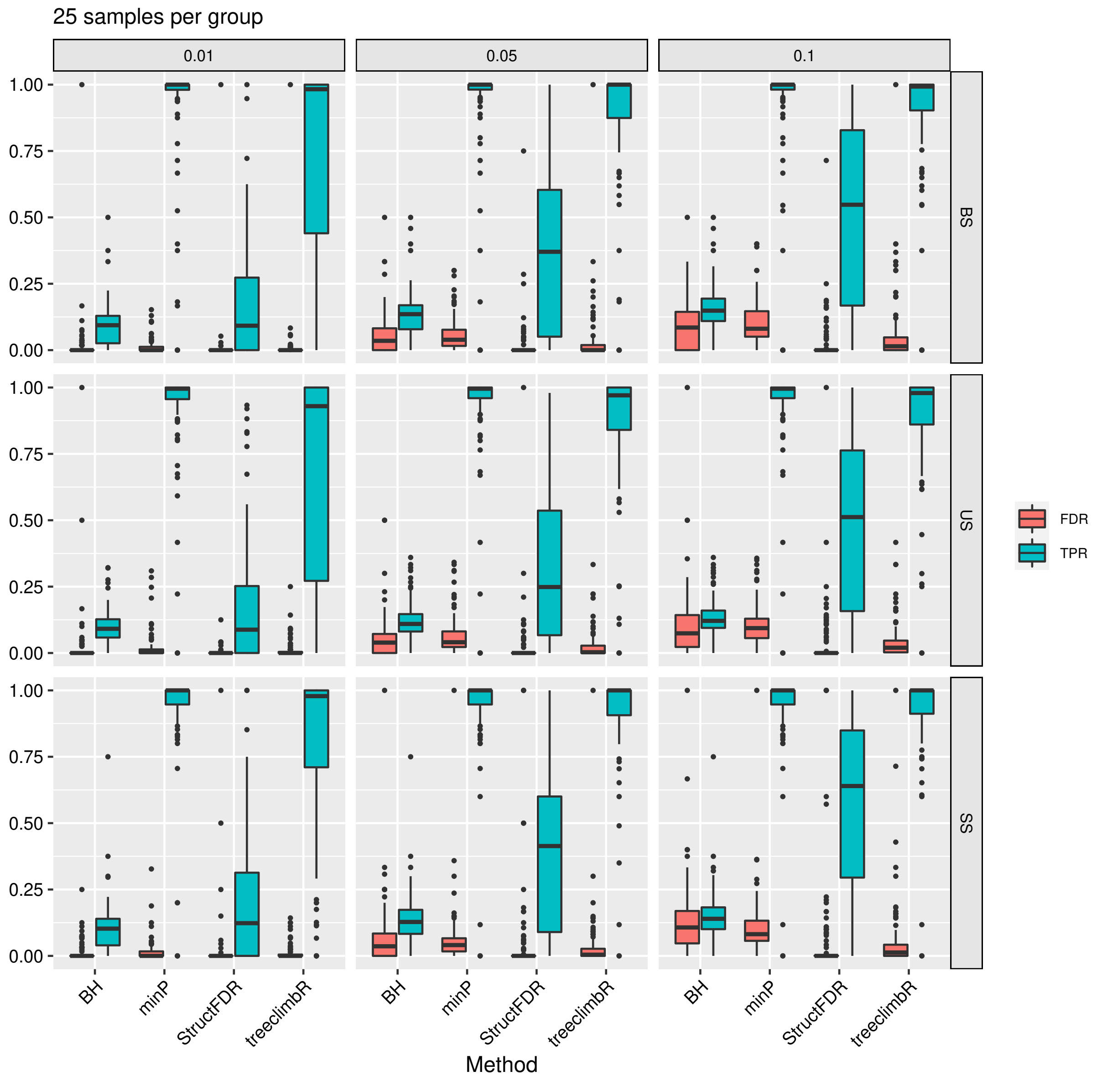

Figure S13. Boxplots of FDR and TPR for four methods on parametric simulated microbial data. Results are across three simulated scenarios (BS, US, and SS) with imposed FDR cutoffs at 0.01**,** 0.05**, and** 0.1**. Results of *BH* and three top tree-based methods in Fig. S12 (*minP*, *StructFDR*, and *treeclimbR*)) are shown. Results in each scenario are based on 100 repetitions.**

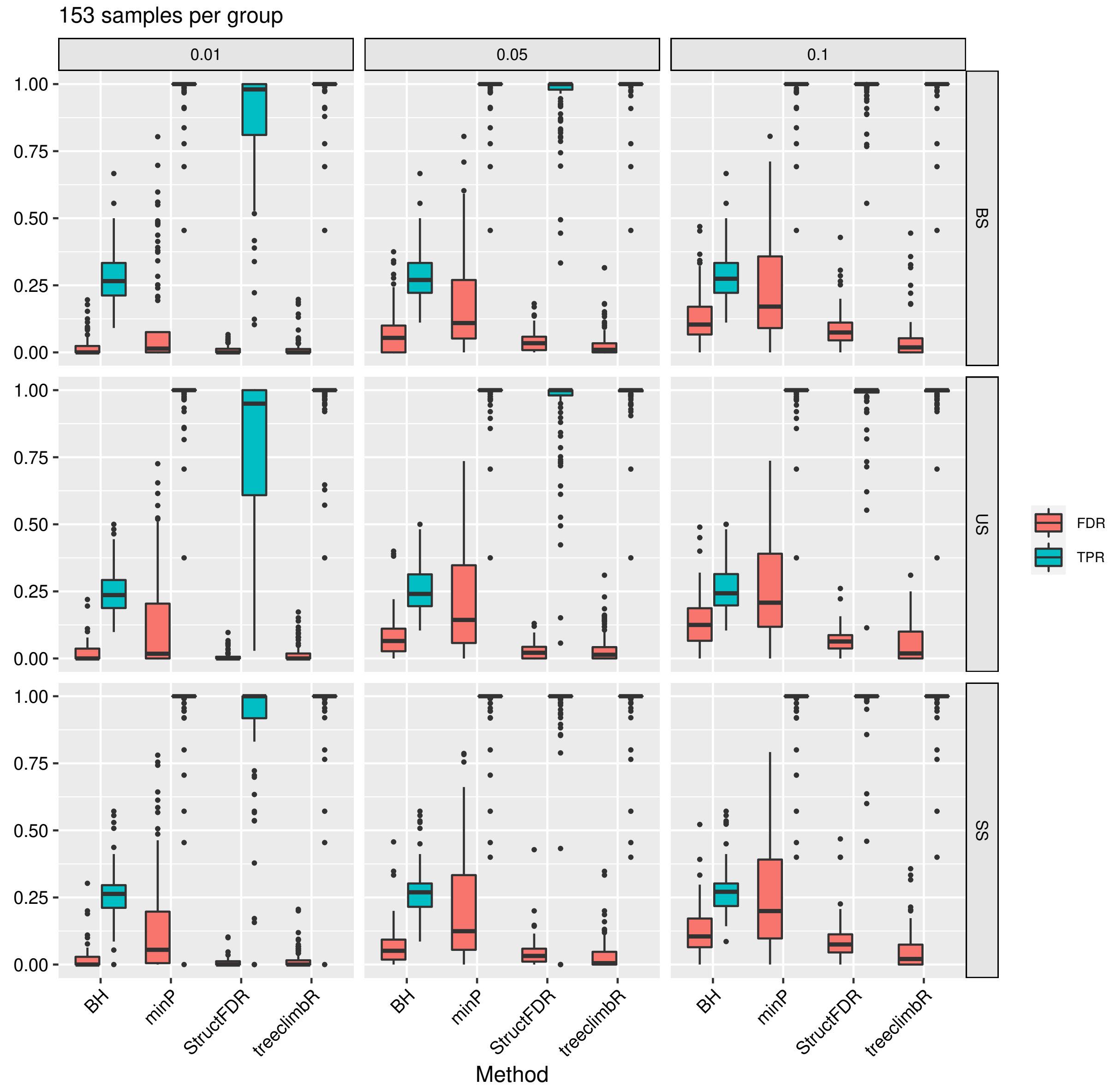

Figure S14. Boxplots of FDR and TPR for four methods on parametric simulated microbial data. Identical to Fig. S13 except that the results are from the simulation with 153 samples per group

Supplementary Note 2:
Details about *treeclimbR*

4.1 The leaf FDR and candidates

Our algorithm *treeclimbR* performs multiple hypothesis tests on the candidate level but controls FDR on the leaf level.

The definition of the leaf FDR. When a null hypothesis on an internal node is rejected, null hypotheses on all its descendant leaves are considered as being rejected too. We define the leaf FDR as the ratio of the number of falsely rejected leaves to the number of rejected leaves. This is formulated as

$$\mathbf{FDR}_L = \mathbf{E}[Q_L] = \mathbf{E}[\frac{V_L}{V_L + S_L}] \tag{5}$$

where V_L and S_L represent the numbers of false positive and true positive leaves, respectively. A true positive leaf is a leaf with non-true null hypothesis and correctly declared as non-true null hypothesis; a false positive leaf is a leaf with true null hypothesis but wrongly declared as non-true null hypothesis;

Examples of candidates. In *treeclimbR*, multiple candidates are obtained by tuning a parameter t to capture the signal pattern on a tree. A toy example with three candidates is shown in Fig. S15. Without using a tree, there are K hypothesis tests on K entities (leaves $1 \sim K$). When a tree is available, *treeclimbR* tends to represent branches that have coherent change with internal nodes. This leads to less hypothesis tests. An example is candidate 2 that replaces leaves $5 \sim 9$ with an internal node X. When t is set too high, candidate 3 that replaces leaves $5 \sim 10$ with an internal node Y might be obtained. We perform multiplicity correction on the candidate level, but aim to control FDR on the leaf level (Equation 5).

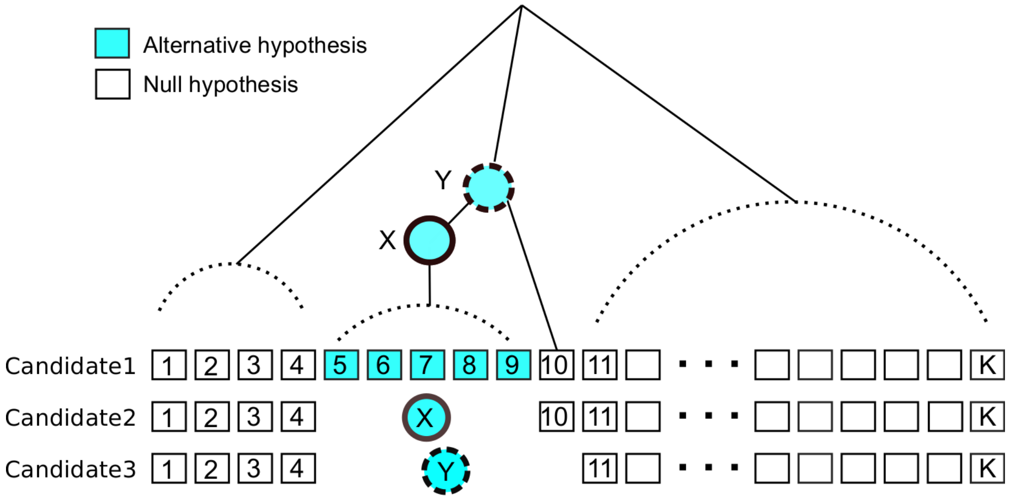

Figure S15. A toy example of three different candidates on the tree. Dashed lines are used to represent branches with different possible structures. Each leaf represents an entity. Entities with true alternative hypothesis are colored in blue; otherwise in white. Node Y is considered as true H_1 in candidate 3 because it includes leaves with true H_1 and its P-value wouldn't follow uniform distribution anymore. The rejection of null hypothesis on node Y would contribute four true positives and one false positive in calculating the leaf FDR. Note: Directions of blue leaves are the same.

The use of candidates might improve or deteriorate the control of the leaf FDR. We know that FDR is defined as Equation 6 in the BH procedure [3].

$$\mathbf{E}[Q] = \mathbf{E}[\frac{V}{V+S}] \tag{6}$$

where V is the number of false positives (true null hypotheses but declared as non-true null hypotheses) and S is the number of true positives (non-true null hypotheses and correctly declared as non-true null hypotheses). When the power to detect signal leaves is the same for both candidates, S of candidate 2 is lower than that of candidate 1 due to the use of internal nodes. Hence, when the FDR is well controlled in both candidates, V in candidate 2 tends to be lower than candidate 1, which implies the leaf FDR is improved in candidate 2. However, it doesn't mean that candidates using internal nodes to represent signal entities always have lower FDR than the leaf candidate. The generation of candidates might introduce new false positives. For example, if null hypothesis on node Y is rejected, the leaf 10 is falsely reported as positive. Such a false positive is caused by the generation of the candidate instead of the BH procedure, and is more likely to occur on candidates generated with high t. To address the issue, the range of t needs to be limited to control the leaf FDR.

4.2 The range of t

A tree might have multiple signal branches, each of which has different surroundings (e.g. the size of sibling branch). To simplify, let's consider one signal branch at a time. In Fig. S15, the node Y is selected to represent leaves $5 \sim 10$ if all its descendants have P-values below the specified threshold t, and the leaf 10 occurs to have the same estimated direction as leaves $5:9$. Generally, the two descendant branches of node Y could be in any configuration (e.g., more leaves or nodes are connected in different ways), and Fig. S15 shows one possible structure.

Let's say the two children of Y are node X and Z. We use $\tilde{\mathbf{H}}$ to represent the fact that all descendant leaves of X are true H_1 and all descendant leaves of Z are true H_0 in Equation 7. Given $\tilde{\mathbf{H}}$, the probability (π) to reject null hypothesis on node Y (H_Y) in a candidate $\mathbf{C(t)}$ could be formulated according to Equation 7.

$$\begin{aligned}
\pi &= \mathbf{Pr}[\{H_Y \notin H_0\} \cap \{Y \in \mathbf{C(t)}\} | \tilde{\mathbf{H}}] && (7)\\
&\leq \mathbf{Pr}[Y \in \mathbf{C(t)} | \tilde{\mathbf{H}}] && (8)\\
&= \mathbf{Pr}[(\cup_{i \in B(Y)} p_i \leq t) | \tilde{\mathbf{H}}] \cdot P^* && (9)\\
&\leq \mathbf{Pr}[(\cup_{i \in b(Y)} p_i \leq t) | \tilde{\mathbf{H}}] \cdot P^{**} && (10)\\
&< \mathbf{Pr}[(\cup_{i \in b(Z)} p_i \leq t) | \tilde{\mathbf{H}}] \cdot P^{**} && (11)\\
&= \prod_{i \in b(Z)} 0.5 \cdot \mathbf{Pr}[p_i \leq t | \tilde{\mathbf{H}}] && (12)\\
&= (0.5t)^n
\end{aligned}$$

where $\mathbf{C(t)}$ is the candidate generated at t; $B(Y)$ includes node Y and its descendant nodes; $b(X)$, $b(Y)$ and $b(Z)$ correspond to descendant leaves of node X, Y and Z; P^* and P^{**} are probability that the estimated directions are the same for nodes in $B(Y)$ and $b(Y)$, respectively; p_i is the P-value of node i; n is the number of descendant leaves of node Z. Equation 7 is less than Equation 8 because the latter doesn't require H_0 on node Y to be

rejected. As discussed above, node Y is selected when it and its descendants agree on the estimated direction and their P-values are below t. So, Equation 8 is equal to Equation 9. Equation 9 is less than Equation 10 because the latter imposes restriction on less nodes ($b(Y) \subseteq B(Y)$). Similarly, Equation 10 is less than Equation 11 because of $b(Z) \subset b(Y)$. As node $i \in b(Z)$ has true H_0, its P-value (p_i) follows uniform distribution ($p_i \sim U(0,1)$) and its probability to show the same estimated direction as leaves of X is 0.5, which means Equation 11 is equal to Equation 12. Hypothesis tests on the leaf level are considered to be independent, so we could further simplify Equation 12 to $(0.5t)^n$, which decreases as n increases at a fixed t. In other words, it becomes unlikely to select and further reject an internal node that represents a signal branch together with a sibling branch when the sibling branch is large. However, once this occurs, it brings more false positives.

The expected number of false positives that are introduced by the generation of candidates depends on the tree structure. A tree that has small non-signal branches next to signal branches is expected to have higher false positives than a tree that have large non-signal branches next to signal branches. Consider a tree that has N branches with signal, the expected number of false positives ($\mathbf{E}[F]$) is maximized in the situation where each signal branch has a one or two-leaf sibling branch that has no signal. Following Equation 12, we have

$$\begin{aligned} \mathbf{E}[F] &= \sum_{i=1}^{N} n_i \cdot \pi_i \\ &< \sum_{i=1}^{N} n_i \cdot (0.5t)^{n_i} \\ &\leq N \cdot 0.5t \end{aligned} \tag{13}$$

where n_i is the number of leaves on the sibling branch of the ith signal branch.

Consider t that is used to generate a candidate, where s_0 true positive nodes (covering l_0 true positive leaves) are reported under the BH [3] procedure with FDR α, as a variable. We would like to get the range of t, in which the leaf FDR is expected to not exceed α. The FDR on the candidate and the leaf level could be formulated as Equation 14 and Equation 15, respectively.

$$\mathbf{E}[Q] = \mathbf{E}[\frac{V}{V + s_0}] \tag{14}$$

$$\mathbf{E}[Q_L] = \mathbf{E}[\frac{V_L}{V_L + l_0}] \tag{15}$$

Here, V and V_L are the number of false positives on the candidate and leaf level, respectively. With a fixed l_0, we could apply Jensen's inequality on Equation 15 to get its upper bound as Equation 16.

$$\begin{aligned} \mathbf{E}[Q_L] &= \mathbf{E}[1 - \frac{l_0}{V_L + l_0}] \\ &\leq 1 - \frac{l_0}{\mathbf{E}[V_L] + l_0} \end{aligned} \tag{16}$$

The number of false positives (V_L) on the leaf level comes from three different sources.

$$\mathbf{E}[V_L] = \mathbf{E}[V_L' + V_L'' + V_L'''] \tag{17}$$

- V'_L: False positives due to the BH [3] procedure performed on the candidate level.
- V''_L: False positives from the generation of a candidate (See candidate 3 in Fig. S15).
- V'''_L: False positives from a case similar to candidate 3 in Fig. S15) but the branch below node X in Fig. S15 has fake signal. In other words, the consistent signal pattern on branch below X is by chance.

Overall, V'_L is due to the procedure of multiple testing correction, V''_L is due to the generation of candidates, and V'''_j is due to the combination of both.

With a fixed s_0, according to Jensen's inequality, the upper bound of Equation 14 is as below.

$$\begin{aligned}\mathbf{E}[Q] &= \mathbf{E}[\frac{V}{V+s_0}] \\ &= 1-\mathbf{E}[\frac{s_0}{V+s_0}] \\ &\leq 1-\frac{s_0}{\mathbf{E}[V]+s_0}\end{aligned}$$

We set the right side of the inequality below α to conservatively control FDR for a candidate.

$$1-\frac{s_0}{\mathbf{E}[V]+s_0} \leq \alpha \Leftrightarrow \mathbf{E}[V] \leq \frac{\alpha}{1-\alpha}s_0 \tag{18}$$

So, the upper bound of false positives allowed in the multiple testing correction is $\frac{\alpha}{1-\alpha}s_0$. According to Equation 13 and Equation 17, we know that

$$\mathbf{E}[V'_L] = \mathbf{E}[V] \leq \frac{\alpha}{1-\alpha}s_0 \tag{19}$$

$$\mathbf{E}[V''_L] \leq \frac{s_0 t}{2} \tag{20}$$

$$\mathbf{E}[V'''_L] \leq \frac{\alpha}{1-\alpha}s_0\frac{t}{2} \tag{21}$$

Therefore,

$$\begin{aligned}\mathbf{E}[V_L] &= \mathbf{E}[V'_L]+\mathbf{E}[V'''_L]+\mathbf{E}[V''_L] \\ &\leq \frac{\alpha}{1-\alpha}s_0+\frac{\alpha}{1-\alpha}s_0\frac{t}{2}+\frac{s_0 t}{2} \\ &= \frac{\alpha s_0}{1-\alpha}+\frac{s_0 t}{2(1-\alpha)}\end{aligned} \tag{22}$$

By substituting Equation 22 to Equation 16, we get

$$\mathbf{E}[Q_L] < 1 - \frac{l_0}{\frac{\alpha s_0}{1-\alpha} + \frac{s_0 t}{2(1-\alpha)} + l_0}$$

$$= \frac{2\alpha + t}{2\alpha + t + 2(1-\alpha)\frac{l_0}{s_0}} \tag{23}$$

$$\tag{24}$$

To control the leaf FDR, we could conversely restrict the upper bound of $\mathbf{E}[Q_L]$ to be below α.

$$\frac{2\alpha + t}{2\alpha + t + 2(1-\alpha)\frac{l_0}{s_0}} \leq \alpha \tag{25}$$

This is further simplified as

$$t \leq 2\alpha(\frac{l_0}{s_0} - 1)$$

To summarize, t is non-negative and its range should be

$$t \in [0, 2\alpha(r-1)]$$

where $r = \frac{l_0}{s_0}$ is the average size of signal branches that are detected. If $r = 1$, signal detected does not cluster on the tree and the leaf level ($t = 0$) should be used to perform multiple test correction. In reality, l_0 and s_0 is unknown and we estimate r as

$$\hat{r} = \frac{l}{s} \tag{26}$$

where s is the number of rejected nodes on the candidate level and l is the number of descendant leaves of rejected nodes. As false positives are less likely to cluster, r tends to be underestimated using $\hat{r}$. This would lead to conservative results on the FDR control.

4.3 The selection of P-values is unbiased in branches without signals

To control FDR on the candidate level when applying the BH procedure, it requires that P-values of true null hypotheses on the candidate level still follow a uniform distribution $[0, 1]$. In other words, the generation of candidates would not lead to a biased selection of P-values on branches without signals. To show this, we generate a negative control case with a tree that has no signal branches.

The toy data includes a random tree with 1000 leaves and a count matrix with 1000 rows and 60 columns. Each row of the matrix is linked to a leaf of the tree and represents an entity. Each column represents a sample. Counts of entities in a sample are randomly taken from a multinomial distribution as below:

$$Y_j \sim \text{Mult}(10000, p)$$

where $p = (p_1, p_2, ..., p_{1000})$ includes the proportions of entities. It is randomly generated and is the same for all samples.

To run *treeclimbR*, candidates are generated using the default values of t in our *treeclimbR* package. The cumulative distribution of P-values on each candidate is shown in Fig. S16, curves of all candidates almost overlap each other, and are very close the dashed line that

represent a uniform distribution in $[0, 1]$, which implies there is no bias in the selection of P-values on different candidates.

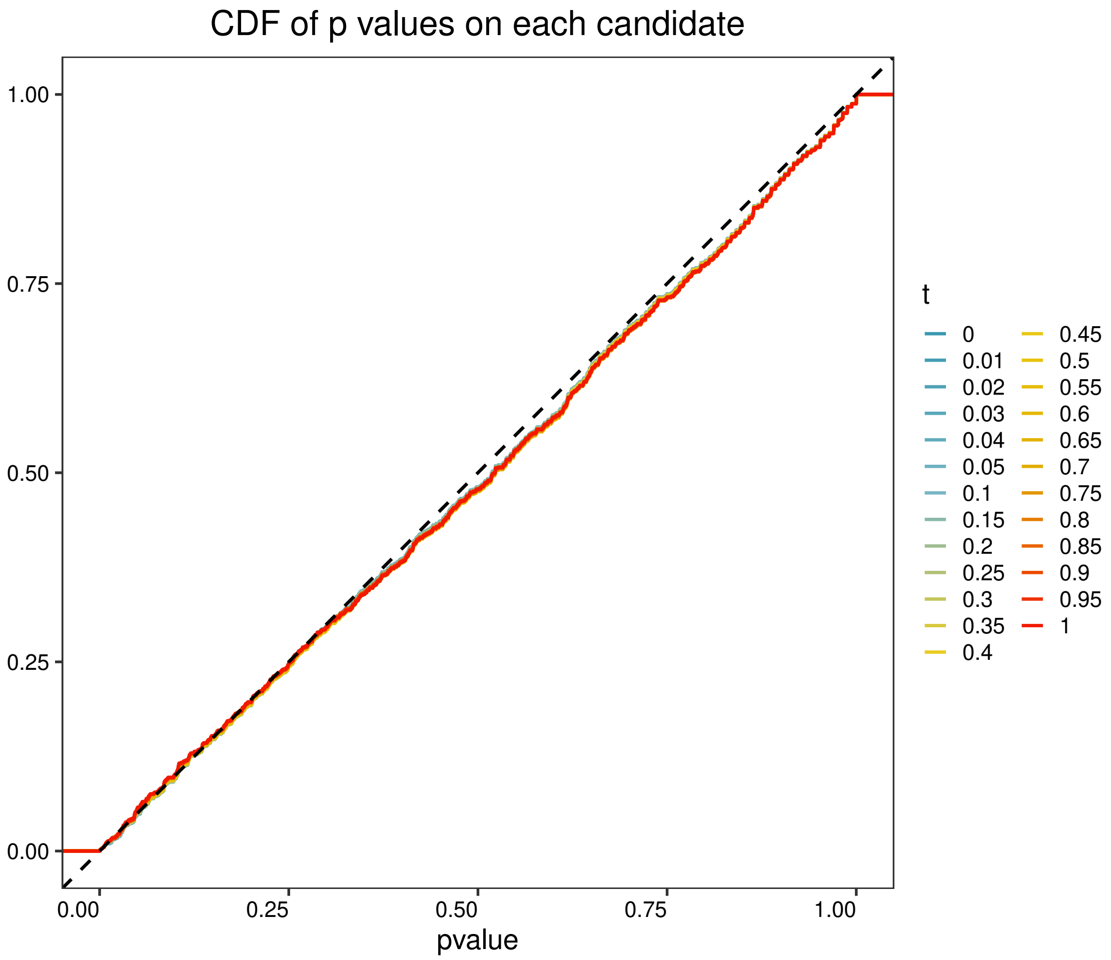

Figure S16. The cumulative distribution of P-values on 25 candidates. Candidates that are generated under different t values have almost the same cumulative distribution. The dashed line represent an uniform distribution in $[0, 1]$.

4.4 An example on toy data

Here, we use a toy dataset to show how *treeclimbR* scans the tree across different t values. In our *treeclimbR* package, 25 values of t in the range $[0, 1]$ are chosen from our experience.

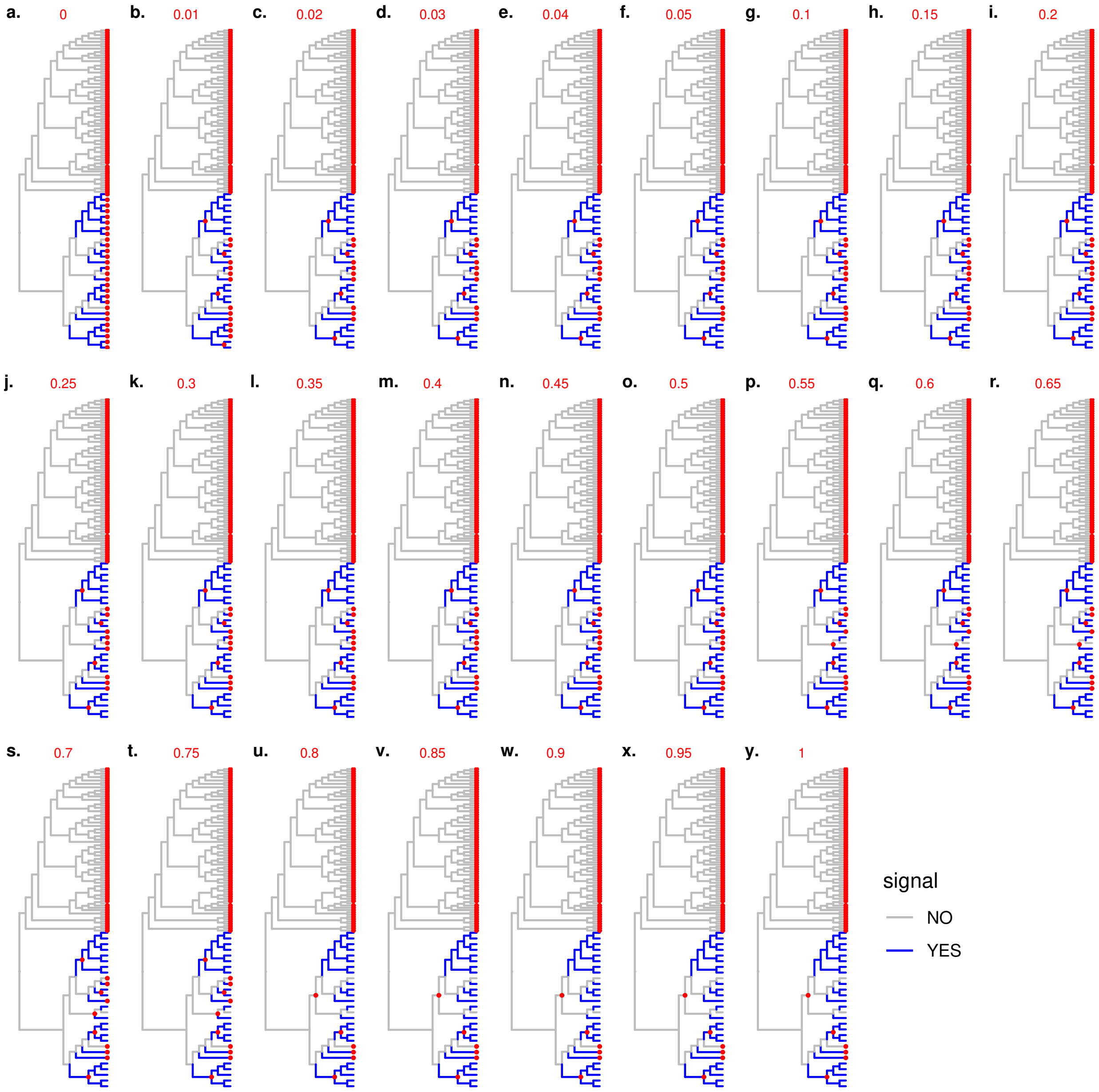

Figure S17. Candidates generated by *treeclimbR* at different t values. DA branches are colored in blue; otherwise in grey. Nodes that are picked to form a candidate are in red points. The t value used for each candidate is given in the title.

The toy data includes a randomly generated tree with 100 leaves and a count matrix with 100 rows and 40 columns. Each row of the count matrix is matched to a leaf. Each column of the count matrix represents a sample. Counts of leaves within a sample are randomly generated from a multinomial distribution with the total count 1000. The probabilities of leaves are generated randomly from a uniform distribution and scaled to have the sum equal to 1. The first 20 samples (or columns) are labeled as the first group, and the other 20 samples as the second group. We further multiply counts of 25 randomly selected leaves by 3 in the second group. In other words, only 25 leaves are different between groups.

treeclimbR is applied on this toy data, and candidates generated at the default values of t in our *treeclimbR* packages are shown in Fig. S17. When $t = 0$, the candidate is on the leaf level. As t increases, multiple DA leaves are represented by internal nodes. When $t = 0.02$, the signal pattern on the tree is captured perfectly. Even when t is further increased, the candidate stays in the same level until $t = 0.55$. In non-DA branches, leaves are picked even at $t = 1$.

Supplementary Note 3: The *minP* procedure

minP was our initial idea to identify signal branches. It includes three main steps: data aggregation, differential analysis, and node selection. The first two steps are the same as in *treeclimbR*. Data on internal nodes are generated from that on leaves, and differential analysis is performed on all nodes of the tree for each feature as

$$\mathbf{p}^g = (p_1^g, p_1^g, ..., p_M^g); \quad g \in (1, 2, ..., G) \tag{27}$$

where M is the total number of nodes on the tree and g represents a feature. Hypotheses on all nodes and features are pooled, and multiplicity correction is performed using the *BH* [3] procedure. Nodes with null hypotheses rejected are collected as a set, S_1. The final step is based on P-values.

1. It starts from the root and moves toward leaves along edges.
2. For each path, it stops when a node i has $p_i \leq p_k$ where node k belongs to descendants of node i ($k \in D(i)$). Nodes that are termini of paths are collected as a set, S_2.
3. Finally, nodes in both S_1 and S_2 are selected.

In other words, a node is selected by *minP* only when its null hypothesis is rejected and none of its descendants have lower P-value.

Supplementary References

[1] Bichat, A., Plassais, J., Ambroise, C. & Mariadassou, M. Incorporating Phylogenetic Information in Microbiome Differential Abundance Studies Has No Effect on Detection Power and FDR Control. *Frontiers in Microbiology* **11**, 649 (2020).

[2] Brito, I. L. *et al.* Mobile genes in the human microbiome are structured from global to individual scales. *Nature* **535**, 435–439 (2016). URL http://www.nature.com/articles/nature18927.

[3] Benjamini, Y. & Hochberg, Y. Controlling the False Discovery Rate: a Practical and Powerful Approach to Multiple Testing. *Journal of the Royal Statistical Society: Series B* **57**, 289–300 (1995).
