# Supplementary material for: treeclimbR pinpoints the data-dependent resolution of hierarchical hypotheses
Source: Genome Biol. 2021 May 17;22:157. doi: 10.1186/s13059-021-02368-1 (PMC8127214; doi:10.1186/s13059-021-02368-1)
Supplement: Supplementary file 3 — Additional file 3 Review history [file 13059_2021_2368_MOESM3_ESM.docx]

**1^st^ round**

**Reviewer 1**

The authors propose treeclimbR, an approach that selects the resolution of hypotheses to test in tree-structured data based on a score statistic combining the P-values and directions of effect for all child nodes. It is a flexible method that could yield interesting biological insights. My comments are as follows:

1. Tree-structured lasso methods have been moderately well developed for continuous outcomes/phenotypes (e.g., "Rare Feature Selection in High Dimensions," JASA Theory and Methods, 2020, Xiaohan Yan and Jacob Bien), although I haven't seen comparable methods for binary outcomes/groups. It may still be worth mentioning these alongside, e.g., Citrus (and briefly explaining why they don't apply in your situation).

2. I have several questions about the microbiome simulations.

a. Why only 5 repetitions for each combination? This does not seem like nearly enough to make well-supported claims about differences in power and type 1 error (or true and false positive rates).

b. All of the sample sizes in the simulated microbiome data are small, whereas the data application is on a fairly large dataset (464 metaOTUs and 381 samples). Does the relative performance of methods change when n = 100, 200, or even larger? (I imagine, for example, that lasso would perform much better with a larger sample size than with n=10-50 per group.)

c. Are branches selected for swapping completely randomly without respect to relative abundance on that branch, number of leaf nodes, etc.? Do you observe differences in performance depending on particular characteristics of the two selected branches? (From line 449 it looks like you kept the same selected signal branches for all five repetitions. It seems as though results could vary quite a bit if signal branches varied. I would feel more confident in the results if this were explored.)

3. How computationally efficient or expensive is treeclimbR? Please provide some computation time benchmarks.

4. In general, I'm in favor of figures whenever possible - but for figures 2b and 3a, a table might be helpful. I'm finding the multitude of lines in 2b difficult to visually interpret without careful study, and in 3a, the overlapping points in some panels make them difficult to interpret.

5. I may have missed this, but in your description of the methods (particularly with respect to the real datasets, but also for simulations), it's not clear to me what tests were performed to generate the node-specific P-values p_k.

6. For non-microbiome datasets, when there are multiple features (rather than only relative abundance), do you perform multiplicity correction on all candidates of all features simultaneously?

7. Supplementary Figure 3: Please explain what all six methods are.

8. In Supplementary Note 1, section 2.2, should t instead be b? I don't see any t in equations (2) or (3).

9. Supplementary Note 2, section 3.3 seems important. I don't see it mentioned in the main text; I'd recommend explicitly discussing it.

10. Supplementary Figure 8: It's a nice demonstration - but the description says the tree stays the same until t=0.3, whereas I don't see any changes until t=0.55. Am I missing something? If there's a convenient way to point out changes, that could be helpful.

**Reviewer 2**

General remarks

Overall, the paper presents an interesting idea and is well-written. The method described, treeclimbR, is concise and flexible. The authors present convincing evidence that the method performs well on both simulated and real data. The authors also carefully explained the assumptions required for treeclimbR and provided helpful notes for applying it in the real-world. The comparisons of treeclimbR to other methods are well visualized and are convincing.

Major points

1. As the authors mentioned in the introduction (line 92), HFDR cannot control global FDR over all features in DS. However, it is not clear how treeclimbR is able to achieve this. It seems DS was treated as G separate DA analyses, and treeclimbR was run for each DA. Was an adjusted p-value used in each DA?

2. The authors compared their methods to many existing ones. However, as they mentioned in the methods sections, some of these methods use different tests to generate p-values in the differential analysis step. Although the choice of tests should not affect the comparisons among methods that takes p-value as input, it might be difficult to interpret the comparisons to others (like lasso).

3. The tree building seems to be a very important step in treeclimbR, as it affects both the interpretation and FDR control. The authors also provided some suggestions in the discussion section. For single cell data, are there any suggestions to how to construct a tree if the goal is to identify new subpopulations that haven't been characterized before? In addition, it would be very interesting to see some example(s) to show how badly the FDR maybe inflated with "double dipping"

4. Related to the point above, the trees in Figure 2C seem to be different from Figure 2A. Are these trees (in Fig.2C) data-driven or pre-defined?

Minor points

1. In the simulations, do the authors have specific considerations when they chose the number of repetitions? It seems the parametric microbial data has 5 repetitions, but AML-sim and BCR-XL-sim only have one. The numbers are quite small -- was it because there were too many differential analyses to run for each repetition?

2. Looks like a typo in Figure 3B caption "… in purple. and labels nodes identified by methods…".

3. In line 226 "and compare the abundance of clusters between the healthy and diseased groups for each cluster". Wasn't it a comparison of the cell counts?

4. In the explanation of Supplementary Materials Equation (5), V should be false positive, S true positive.

**Authors’ response**

**Reviewer #1**: The authors propose treeclimbR, an approach that selects the resolution of hypotheses to test in tree-structured data based on a score statistic combining the P-values and directions of effect for all child nodes. It is a flexible method that could yield interesting biological insights. My comments are as follows:

1. Tree-structured lasso methods have been moderately well developed for continuous outcomes/phenotypes (e.g., "Rare Feature Selection in High Dimensions," JASA Theory and Methods, 2020, Xiaohan Yan and Jacob Bien), although I haven't seen comparable methods for binary outcomes/groups. It may still be worth mentioning these alongside, e.g., Citrus (and briefly explaining why they don't apply in your situation).

Tree-structured lasso methods, e.g., (TASSO, and rare) are now added in the introduction, alongside Citrus. We include the general method, lasso, instead of Citrus in the benchmark because the software Citrus is available specifically for Flow/Mass cytometry data and its performance on two semi-simulated CyTOF datasets (AML-sim and BCR-XL-sim) has been shown in the *diffcyt* paper (see Fig.2a. Fig.3a of https://www.nature.com/articles/s42003-019-0415-5 ).

2. I have several questions about the microbiome simulations.

a. Why only 5 repetitions for each combination? This does not seem like nearly enough to make well-supported claims about differences in power and type 1 error (or true and false positive rates).

Five repetitions were initially chosen because some methods in the benchmark take considerable time to finish one repetition. Now, Figure 2 is updated with 100 repetitions.

b. All of the sample sizes in the simulated microbiome data are small, whereas the data application is on a fairly large dataset (464 metaOTUs and 381 samples). Does the relative performance of methods change when n = 100, 200, or even larger? (I imagine, for example, that lasso would perform much better with a larger sample size than with n=10-50 per group.)

Sorry, there was an error about the number of samples, which should be 285 instead of 381 (96 mother samples were not used in our data analysis). This is now corrected in the manuscript. The 285 samples include measurements at three different time points, which means 95 samples in total for two groups at each time point. In our previous simulation, we had already simulated a similar sample size (50 samples per group in Figure 2). In Supplementary Figure 12, we provide results of a simulation with n=153 (the sample size of data ***throat_v35*** that simulation is based on) per group where lasso shows a little better TPR but also higher FDR, and still does not perform as well as methods, e.g., StructFDR or treeclimbR, in such large sample size.

c. Are branches selected for swapping completely randomly without respect to relative abundance on that branch, number of leaf nodes, etc.? Do you observe differences in performance depending on particular characteristics of the two selected branches? (From line 449 it looks like you kept the same selected signal branches for all five repetitions. It seems as though results could vary quite a bit if signal branches varied. I would feel more confident in the results if this were explored.)

In Figure 2, we fixed the selected branches but changed the signal pattern to show how *treeclimbR* captures the different signal patterns (Figure 2c). The two branches were chosen randomly according to size. To show performance of methods on branches with different characteristics, we have added new simulations that vary branches randomly in repetitions and provided results in Supplementary Figures 9-14. Overall, we simulate each scenario (BS, US, SS) on randomly selected branches for 100 repetitions with different sample sizes (25 and 153 samples per group). The new results still show the strong performance of *treeclimbR*.

3. How computationally efficient or expensive is treeclimbR? Please provide some computation time benchmarks.

Benchmarking results of methods' runtimes are now provided in Supplementary Figure 7. It takes about 30 minutes for treeclimbR to run on a tree with 10,000 leaves. The increase of sample size from 100 samples to 500 samples has almost no effect on *treeclimbR*. Some tree-based methods (e.g., miLineage and LEFse) do not accept the tree format directly as the input, which requires additional time in data preparation. To show better performance of competitors, we do not include the time of data preparation in the run time for methods other than treeclimbR and minP. Also, to obtain results for different FDR levels, tree-based methods (miLineage, HFDR, StructFDR) would need to rerun the whole procedure every time. However, treeclimbR, which separates the candidate proposal step and the multiplicity correction step, only needs to find candidates (the most computation intensive step) once, and uses them to correct FDR at different levels.

4. In general, I'm in favor of figures whenever possible - but for figures 2b and 3a, a table might be helpful. I'm finding the multitude of lines in 2b difficult to visually interpret without careful study, and in 3a, the overlapping points in some panels make them difficult to interpret.

Results shown in tables are also overwhelming and not straightforward to retrieve the information. As a compromise, the underlying numbers of the two mentioned figures are provided in Supplementary Tables 2 and 3.

5. I may have missed this, but in your description of the methods (particularly with respect to the real datasets, but also for simulations), it's not clear to me what tests were performed to generate the node-specific P-values p_k.

A new subsection "Differential analysis" is now added in the "Description of treeclimbR methodology" to describe the statistical tests that were performed for all datasets.

6. For non-microbiome datasets, when there are multiple features (rather than only relative abundance), do you perform multiplicity correction on all candidates of all features simultaneously?

In the case of multiple features (e.g., differential state analysis), we let each feature independently climb the tree (***T***) to propose candidates at different ***t***. For a specific ***t,*** the global candidate includes all (sub)-candidates generated for all features (Equation 6 and Supplementary Figure 1). The multiplicity correction is performed within each global candidate as described in Figure 2c, d. We have added new text in the Overview section to explain this.

7. Supplementary Figure 3: Please explain what all six methods are.

We have edited the caption of Supplementary Figure 3 to clarify the six methods, and have also added more details in the Supplementary section 1.3.

8. In Supplementary Note 1, section 2.2, should t instead be b? I don't see any t in equations (2) or (3).

Yes, t is b. The correction is made.

9. Supplementary Note 2, section 3.3 seems important. I don't see it mentioned in the main text; I'd recommend explicitly discussing it.

This is mentioned now at the end of the section "The generation of candidates".

10. Supplementary Figure 8: It's a nice demonstration - but the description says the tree stays the same until t=0.3, whereas I don't see any changes until t=0.55. Am I missing something? If there's a convenient way to point out changes, that could be helpful.

Yes, it should be t=0.55. We have corrected the description (Supplementary Figure 8 becomes Supplementary Figure 17 after the revision).

Reviewer #2: General remarks

Overall, the paper presents an interesting idea and is well-written. The method described, treeclimbR, is concise and flexible. The authors present convincing evidence that the method performs well on both simulated and real data. The authors also carefully explained the assumptions required for treeclimbR and provided helpful notes for applying it in the real-world. The comparisons of treeclimbR to other methods are well visualized and are convincing.

Major points

1. As the authors mentioned in the introduction (line 92), HFDR cannot control global FDR over all features in DS. However, it is not clear how treeclimbR is able to achieve this. It seems DS was treated as G separate DA analyses, and treeclimbR was run for each DA. Was an adjusted p-value used in each DA?

In treeclimbR, the step of candidate proposal and the step of multiplicity correction is separated. Therefore, we can pool the sub-candidates from multiple features to form a global candidate for each ***t*** in the step of candidate proposal, and further control the global FDR at a desired level. HFDR needs the FDR level in its step-down procedure along the tree, which means the search might go to different branches of the tree for different FDR levels. To control the global FDR in the DS case, the HFDR would hence need to first allocate FDR for different features, which is either not possible or at least is currently not available. We have added new text in the Overview section to explain more about treeclimbR in the DS case.

2. The authors compared their methods to many existing ones. However, as they mentioned in the methods sections, some of these methods use different tests to generate p-values in the differential analysis step. Although the choice of tests should not affect the comparisons among methods that take p-value as input, it might be difficult to interpret the comparisons to others (like lasso).

We have added a new subsection *"Differential analysis"* in the *"Description of treeclimbR methodology "* to clarify how P-values were computed for different methods. Overall, all benchmarked methods use the same method to generate the P-values (edgeR, limma or non-parametric Wilcoxon rank sum test according to the dataset) for the same dataset except that *lasso* does not require P-values as input and *miLineage* has its own specified method to compute P-values. Of course, *lasso* does not aim at FDR control but rather is geared towards prediction; we include it here because of its popularity for feature selection on biological data. In many situations, identification of DA or DS entities is of interest instead of prediction. Thus, the FDR and TPR of LASSO are also relevant information for users to know.

3. The tree building seems to be a very important step in treeclimbR, as it affects both the interpretation and FDR control. The authors also provided some suggestions in the discussion section. For single cell data, are there any suggestions to how to construct a tree if the goal is to identify new subpopulations that haven't been characterized before? In addition, it would be very interesting to see some example(s) to show how badly the FDR maybe inflated with "double dipping"

Similar to other tree-based algorithms, the use of treeclimbR assumes the data has the tree structure available for the analysis. Overall, it is important for users to be aware of double dipping. For single cell data, if the main interest is to explore data at high resolution, users can over-cluster cells into many cell subpopulations based on canonical or computational lineage markers. Currently, there are many tools available to cluster single cells into cell subpopulations. Software packages, e.g., Seurat1, scanpy2, and SINCERA3, have provided workflows to prepoccess and cluster cells in unsupervised ways. The over-clustered cell subpopulations can further be arranged as leaves of a tree (e.g., by hierarchical clustering) according to their similarities in profile expressions of features. It is important that the leaf resolution has sufficient data to perform differential analysis, which might depend on the software used to perform differential analysis. Indeed, the tree building affects the interpretation.

To explore the ramifications of double dipping, we ran one example that uses the abundance table to construct the tree and also to perform the differential analysis. The result is provided in Supplementary Figure 5, and shows improved TPR but also inflated FDR when samples from both groups are used in the tree construction.

4. Related to the point above, the trees in Figure 2C seem to be different from Figure 2A. Are these trees (in Fig.2C) data-driven or pre-defined?

Indeed, the trees in Figure 2a and 2c are different. Trees in Figure 2a are toy example trees (randomly generated from the R package ape) to schematically explain the simulated signal patterns. The tree in Figure 2c is from a microbial dataset, which is a real tree with 956 leaves. To visualize such a big tree, we use dashed lines representing branches without DA signals to save space for the display of signal branches. That's why rows of the heatmap corresponding to dashed lines (consisting of many OTUs) are more gold (abundant). We have added new text in the figure caption to clarify this.

Minor points

1. In the simulations, do the authors have specific considerations when they chose the number of repetitions? It seems the parametric microbial data has 5 repetitions, but AML-sim and BCR-XL-sim only have one. The numbers are quite small -- was it because there were too many differential analyses to run for each repetition?

We chose 5 repetitions initially because some methods in the benchmark take considerable time to finish one repetition. For the parametric microbial data, we have now conducted 100 repetitions for each scenario (US, BS, and SS). We have also added new simulations to vary signal branches across repetitions, and results are given in Supplementary Figures 9-14. AML-sim and BCR-XL-sim are from the *diffcyt* article*,* which are both *semi-simulated* datasets and only one repetition is available.

2. Looks like a typo in Figure 3B caption "… in purple. and labels nodes identified by methods…".

We have fixed the typo: purple. → purple, .

3. In line 226 "and compare the abundance of clusters between the healthy and diseased groups for each cluster". Wasn't it a comparison of the cell counts?

This is changed to: "and compare the cell counts of clusters between the healthy and diseased groups for each cluster".

4. In the explanation of Supplementary Materials Equation (5), V should be false positive, S true positive.

Thank you. This error is fixed.

1 Macosko, E. Z. et al. Highly parallel genome-wide expression profiling of individual cells using nanoliter droplets. *Cell* 161, 1202–1214 (2015).

2 Wolf, F. A., Angerer, P. & Theis, F. J. SCANPY: large-scale single-cell gene expression data analysis. *Genome Biol.* 19, 15 (2018).

3 Guo, M., Wang, H., Potter, S. S., Whitsett, J. A. & Xu, Y. SINCERA: a pipeline for single-cell RNA-Seq profiling analysis. *PLOS Comput. Biol.* 11,

e1004575 (2015).

**2^nd^ round**

**Reviewer 1**

Thank you for the additional simulations and explanation - my comments have been addressed.

**Reviewer 2**

All concerns were addressed.
